# Rational design of FAP-targeted sEVs delivered by microneedles for precision treatment of hypertrophic scars via ferroptosis in hypertrophic scar fibroblasts

**DOI:** 10.1016/j.mtbio.2026.103117

**Published:** 2026-04-13

**Authors:** Yangmengyuan Xu, Yiqing Zhang, Zijie Sun, Junfeng Gong, Qi Shen, Hao Meng, Xi Liu, Junli Chen, Yaying Hao, Zhan Xu, Kui Ma, Liqian Ma, Kailu Guo, Xiaohua Pan, Xiaobing Fu, Cuiping Zhang

**Affiliations:** a Medical Innovation Research Department, PLA General Hospital and PLA Medical College, Beijing, 100048, China; b College of Graduate, Tianjin Medical University, Tianjin, 300070, China; c Institute of Clinical Translation and Regenerative Medicine, The Second Affiliated Hospital of Shenzhen University, Shenzhen, 518100, China

**Keywords:** Small extracellular vesicles, Fibroblast activation protein, Ferroptosis, Hypertrophic scars, Dissolvable microneedles

## Abstract

Hypertrophic scars (HS) lack cell-specific therapies because pathogenic hypertrophic scar fibroblasts (HSFs) are hard to inactivate without damaging human dermal fibroblasts (HDFs). Ferroptosis has emerged as a potential antifibrotic vulnerability of activated fibroblasts, yet its translational use is constrained by off-target toxicity of small-molecule inducers. Here, we report a targeted delivery strategy based on small extracellular vesicles (sEVs) surface-decorated with a fibroblast activation protein (FAP) ligand to preferentially deliver a widely used ferroptosis inducer erastin to HSFs. We confirmed that FAP is upregulated in HSFs and HS tissue, and engineered adipose-derived mesenchymal stem cell (ADSC)-derived sEVs loaded with erastin and covalently conjugated with the FAP ligand UAMC1110 (sEVs^ErF^) via EDC/NHS chemistry. sEVs^ErF^ retained typical nanoscale characteristics and EV marker profiles and showed preferential uptake by HSFs over HDFs *in vitro*, which was validated by competitive blocking and FAP knockdown experiments. Functionally, sEVs^ErF^ suppressed HSF proliferation and migration and reduced α-SMA, COL I, and COL III expression, accompanied by ferroptosis-associated signatures including lipid peroxidation, glutathione depletion, labile Fe^2+^ accumulation, and mitochondrial dysfunction. For localized dermal delivery, sEVs^ErF^ were incorporated into dissolvable microneedle patches (DMNPs) with GelMA tips and a PVA backing. In a rabbit ear HS model, sEVs^ErF^-DMNPs reduced scar thickness and collagen deposition and modulated ferroptosis- and fibrosis-related readouts in scar tissues. Both *in vitro* and *in vivo* rescue experiments using the ferroptosis inhibitor Ferrostatin-1 confirmed that these effects were specifically mediated by ferroptosis. Collectively, these results support FAP as an actionable targeting handle for preferential delivery to scar fibroblasts and suggest that ferroptosis-associated inactivation of HSFs can be leveraged through a microneedle-enabled, locally retained sEV formulation.

## Highlights

• FAP-targeted sEVs selectively deliver erastin to hypertrophic scar fibroblasts.
• Microneedle patches enable localized, painless, sustained intradermal sEV delivery.
• sEVs^ErF^ triggers ferroptosis in hypertrophic scar fibroblasts and reduces fibrosis.
• Rabbit ear model shows improved scar elevation index and collagen I/III remodeling.
• Modular FAP-sEV–microneedle platform is adaptable to FAP-associated diseases.

## Graphical abstract

**Figure:**
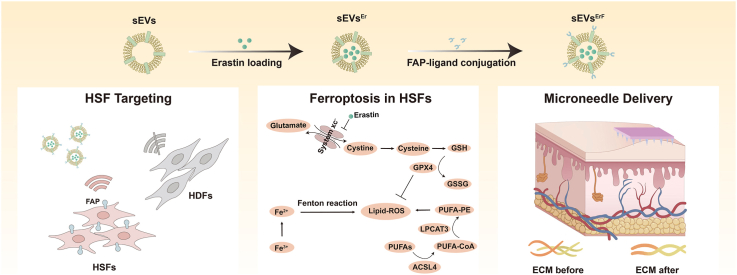

## 1 Introduction

Hypertrophic scars (HS) are pathological outcomes of wound healing, remain a clinical challenge owing to suboptimal therapeutic efficacy and high recurrence, often necessitating repeated interventions [1]. The core cellular mechanisms involve the abnormal activation of fibroblasts and the imbalance of collagen metabolism [2,3]. During the proliferative phase of wound healing, human dermal fibroblasts (HDFs) are activated to proliferate and secrete collagen to accelerate wound closure [4]. In pathological remodeling, HDFs are activated excessively and acquire a myofibroblast-like phenotype, namely hypertrophic scar fibroblasts (HSFs), which are responsible for excessive collagen production and tissue remodeling [5]. Selectively disabling the function of HSFs by targeted intervention strategies can effectively inhibit scar formation without affecting wound healing [6].

Ferroptosis is a non-apoptotic and iron-dependent form of regulated cell death characterized by the accumulation of lipid peroxidation to lethal levels [7]. Ferroptosis activators are expected to be used for the treatment of HS by inducing ferroptosis in HSFs and inhibiting their excessive proliferation and collagen deposition, thereby reducing scar formation [8]. Intriguingly, HSFs are reported to be highly susceptible to ferroptosis, increasing the possibility of ferroptosis activators as therapeutic molecules for HS [9]. Erastin is currently one of the most extensively studied and widely used inducers of ferroptosis by suppressing cystine/glutamate antiporter system Xc^−^ (SLC7A11/xCT), depleting glutathione (GSH), and functionally inactivating glutathione peroxidase 4 (GPX4), ultimately causing lethal lipid peroxide accumulation [10]. However, a major concern in the therapeutic application of erastin is its off-target toxicity toward normal cells [11].

Recently, engineered small extracellular vesicles (sEVs) have garnered attention for targeted delivery of drugs [12,13]. sEVs are typically <200 nm lipid-bilayer nanovesicles secreted by diverse cell types, transporting proteins, nucleic acids, lipids, and small therapeutic agents [14]. Targeting is typically introduced by surface modification with HSF-targeted antibodies, ligands or peptides, which are expected to have specific affinity to surface markers on HSFs [15]. Fibroblast activation protein (FAP) is highly expressed on HSFs and enriched in HS tissue, providing an actionable surface marker for engineering sEVs [16]. UAMC1110, a high-affinity FAP inhibitor, provides a possible strategy for constructing FAP-targeting therapeutic sEVs [17]. 1-ethyl-3-(3-dimethylaminopropyl)carbodiimide/N-hydroxysuccinimide (EDC/NHS) amide coupling is a widely used bioconjugation reaction that forms stable amide bonds between carboxyl groups and primary amines, enabling covalent attachment of targeting ligands to nanocarrier surfaces [18]. Applied to sEV engineering, EDC/NHS chemistry can be used to decorate sEV membranes with HSF-targeting ligands, thereby improving HSF selectivity for ferroptosis activator delivery and reducing off-target injury to normal tissues [19].

To further increase the precision of scar treatment, HSF-targeted sEVs need to be transported to the dermis layer. Currently, multi-injection of sEVs is frequently used for the treatment of wounds and scars, but the shortcomings are also obvious, such as causing great pain to patients and resulting in uneven distribution and low local retention of sEVs [20]. Dissolvable microneedle patches (DMNPs) have recently been developed as transdermal drug delivery devices with efficient, painless, microinvasive, and sustained release properties [21]. Normally, the microneedle patch consists of two parts including ultra-fine tips and a flexible backing base. The superfine tips endow the microneedle patch with strong mechanical strength to form microchannels in the skin and flexible backing base has enough flexibility to fit different bends. Various biocompatible and biodegradable materials, such as photocrosslinked gelatin methacryloyl (GelMA), are suitable for the preparation of microneedle tips, while polyvinyl alcohol (PVA) can offer flexibility for irregular hypertrophic-scar surfaces [22]. Our previous study reported the use of DMNPs to deliver sEVs for HS treatment [23].

In this study, we first compared the expression of FAP in HDFs and HSFs. sEVs isolated from adipose-derived mesenchymal stem cells (ADSCs) were loaded with ferroptosis activator erastin and modified with FAP ligand UAMC1110 by EDC/NHS amide coupling. The structural integrity and HSF-targeted property of sEVs^ErF^ were evaluated. *In vitro*, sEVs^ErF^ inhibited the pathological behaviors of HSFs and activated ferroptosis-related indicators. Subsequently, sEVs^ErF^ were encapsulated in DMNPs for HS treatment. *In vivo*, sEVs^ErF^-DMNPs effectively reduced the thickness of HS and collagen deposition, which was correlated with the downregulated HSF functions induced by ferroptosis.

## 2 Materials and methods

### 2.1 Materials

N-Boc-PEG_3_-carboxylic acid, 4-quinolinecarboxamide derivative, Fmoc-Lys (Ac) and succinic anhydride were purchased from Hefei Taikubio Co., Ltd. (Hefei, China). HATU, HOBt, DIEA, NMM, TEA, anhydrous N,N-dimethylformamide (DMF), trifluoroacetic acid (TFA), piperidine and diethyl ether were also obtained from Hefei Taikubio Co., Ltd. Erastin was purchased from MedChemExpress (Monmouth Junction, NJ, USA). EDC, NHS and MES were obtained from Aladdin (Shanghai, China). GelMA, PVA and the 405 nm photopolymerization device were purchased from Engineering For Life (Suzhou, China). Phosphate-buffered saline (PBS) was purchased from Beyotime Biotechnology (Shanghai, China). All chemicals were of analytical grade.

### 2.2 Cells and animals

ADSCs and complete ADSC medium were obtained from OriCell (Shanghai, China). HSFs and the corresponding primary fibroblast culture system were purchased from iCell Bioscience Inc. (Shanghai, China) and maintained according to the manufacturer's instructions. HDFs were obtained from primary cultures previously established by our group, as described in our earlier work [23].

Male New Zealand white rabbits (2.5-3.0 kg) were provided by Beijing Keyu Experimental Animal Breeding Center (Beijing, China). All animal procedures were approved by the Animal Welfare and Ethics Committee of Beijing Keyu Experimental Animal Breeding Center (approval code: KY20250310006) and conducted in accordance with institutional and national guidelines. Rabbits were housed under standard conditions with free access to food and water.

### 2.3 Isolation and identification of sEVs

ADSCs were cultured in complete medium at 37 °C with 5% CO_2_ until reaching 70-80% confluence. For sEV collection, cells were washed with PBS and incubated in serum-free medium for 24 h. The culture supernatant was harvested and cleared by sequential centrifugation to remove cells and debris (300×*g* for 10 min, 2000×*g* for 10 min, and 10,000×*g* for 30 min, 4 °C). The supernatant was then passed through a 0.22 μm membrane filter to further remove residual particulates. sEVs were isolated by differential ultracentrifugation at 100,000×*g* for 75 min at 4 °C using an ultracentrifuge (Optima XPN-100, Beckman Coulter, USA). The pellet was resuspended in sterile PBS and ultracentrifuged again at 100,000×*g* for 75 min at 4 °C for washing. Purified sEVs were finally resuspended in PBS, aliquoted, and stored at 4 °C for no longer than 48 h or at −80 °C for long-term preservation, avoiding repeated freeze-thaw cycles. Unless otherwise stated, EV-related terminology follows MISEV2018, and we use “sEVs” to describe <200 nm extracellular vesicles enriched by the applied isolation workflow.

The morphology of sEVs was examined by transmission electron microscopy (TEM; HT7800, Hitachi, Japan). Particle size distribution and concentration were determined by nanoparticle tracking analysis (NTA; Particle Metrix, Germany). The expression of sEV-associated markers CD9, CD63 and TSG101 and the absence of the endoplasmic reticulum protein Calnexin were confirmed by Western blotting using antibodies against CD9 (13,174, Cell Signaling Technology, USA), CD63 (ab134045, Abcam, UK), TSG101 (72,312, Cell Signaling Technology, USA) and Calnexin (ab22595, Abcam, UK).

### 2.4 Synthesis of SUC-Lys (Ac)-PEG_3_-UAMC1110 and SUC-Lys (FITC)-PEG_3_-UAMC1110

SUC-Lys (Ac)-PEG_3_-UAMC1110 was synthesized by stepwise amide coupling starting from N-Boc-PEG_3_-carboxylic acid and a 4-quinolinecarboxamide derivative. Briefly, N-Boc-PEG_3_-carboxylic acid (1.0 eq) was dissolved in anhydrous DMF and activated with HATU and DIEA at room temperature for 5 min. The 4-quinolinecarboxamide derivative (1.0 eq) was then added, and the mixture was stirred for 1-2 h to give crude compound 3 (Boc-protected intermediate). After solvent removal under reduced pressure, the residue was treated with TFA and precipitated with diethyl ether. The precipitate was collected by centrifugation, dried under vacuum, and purified by reverse-phase HPLC to afford compound 3. Fmoc-Lys (Ac) (compound 4) was dissolved in DMF, activated with EDC and HOBt at 0 °C for 10 min, and then reacted with compound 3 in the presence of NMM. The reaction was allowed to proceed for 2 h at room temperature. After solvent evaporation, the residue was treated with 20% piperidine in DMF to remove the Fmoc group, washed with diethyl ether, and purified by reverse-phase HPLC to obtain compound 5. Finally, compound 5 was reacted with succinic anhydride (compound 6) and DIEA in DMF for 2 h at room temperature. The crude product was purified by reverse-phase HPLC to yield SUC-Lys (Ac)-PEG_3_-UAMC1110. The identity and purity of the product were confirmed by analytical HPLC and MS.

SUC-Lys (FITC)-PEG_3_-UAMC1110 was prepared using an analogous strategy. Compound 3 was first obtained as described above. After coupling of compound 3 with Fmoc-Lys (Ac) (compound 4) and subsequent Fmoc deprotection, the resulting intermediate (compound 5) was purified by reverse-phase HPLC. Compound 5 was then dissolved in DMF and reacted with succinic anhydride (compound 6) in the presence of DIEA at room temperature for 2 h. After brief treatment with TFA and precipitation with cold diethyl ether, the crude product was purified by reverse-phase HPLC to afford the succinylated intermediate (compound 7). Compound 7 was then dissolved in DMF and reacted with FITC in the presence of TEA at room temperature for 2 h. The reaction mixture was purified by reverse-phase HPLC to give SUC-Lys (FITC)-PEG_3_-UAMC1110. The final product was characterized by analytical HPLC and MS.

### 2.5 Preparation of erastin-loaded and FAP-targeted sEVs

A full-wavelength UV-Vis scan of erastin (12.5-800 μM) confirmed a maximal absorbance at 280 nm. An A280 calibration curve (0-800 μM) was generated using erastin standards prepared in lysed sEVs under the same lysis conditions as the samples. For drug loading, erastin (25-400 μM) was mixed with ∼1.0 × 10^10^ sEVs and incubated at 37 °C for 1 h. Free erastin was removed by ultracentrifugation (100,000×*g*, 70 min, 4 °C), the pellet was washed twice with PBS and re-pelleted. The resulting sEV samples were lysed by mixing 1:1 (v/v) with RIPA lysis buffer, incubating on ice for 30 min, and centrifuging to remove insoluble debris. The absorbance at 280 nm (A280) was then measured, the A280 from blank sEV lysates was subtracted, and the net A280 was converted to the concentration of encapsulated erastin using the calibration curve. The resulting erastin-loaded vesicles are referred to as sEVs^Er^.

To generate FAP-targeted formulations, SUC-Lys (Ac)-PEG_3_-UAMC1110 or SUC-Lys (FITC)-PEG_3_-UAMC1110 was coupled to sEVs^Er^ by carbodiimide chemistry. Ligands were activated in 50 mM MES buffer (pH 5.5) with EDC and NHS (ligand:EDC:NHS ≈ 1:1.5:1) for 15-30 min at room temperature, added to sEV suspensions in PBS (pH 7.4) and incubated overnight at 4 °C with gentle agitation. Excess ligands were removed by ultracentrifugation (100,000×*g*, 70 min, 4 °C). The resulting FAP-targeted, erastin-loaded vesicles are denoted sEVs^ErF^. For fluorescent formulations, SUC-Lys (FITC)-PEG_3_-UAMC1110 was used, and conjugation efficiency and ligand density were quantified on a Flow NanoAnalyzer (N30E, NanoFCM, China) from FITC fluorescence and particle counts using an external FITC calibration curve.

For assessing the targeting ligand alone, FAP-targeted sEVs without erastin loading (sEVs^F^) were prepared using the same EDC/NHS conjugation procedure with SUC-Lys (Ac)-PEG_3_-UAMC1110, followed by ultracentrifugation (100,000×*g*, 70 min, 4 °C) to remove excess ligand.

### 2.6 *In vitro* uptake and targeting assays of sEVs^ErF^

*In vitro* uptake and targeting of sEV formulations were evaluated in HSFs and HDFs. FITC-labeled sEVs were generated by coupling SUC-Lys (FITC)-PEG_3_-UAMC1110 to the vesicle surface as described above, so that green fluorescence reported successful FAP-ligand display. For cell-type specificity analysis, HSFs and HDFs were incubated with FITC-labeled sEVs for 2 h at 37 °C. For time-course uptake analysis, HSFs were incubated with FITC-labeled sEVs^ErF^ for 0.5, 2, 6, and 12 h at 37 °C. For competitive blocking experiments, HSFs were pretreated with free UAMC1110 (1 μM, HY-100684, MedChemExpress, USA) for 1 h at 37 °C prior to the addition of FITC-labeled sEVs^ErF^, followed by 2 h incubation.

After incubation, cells were washed with PBS, fixed with 4% paraformaldehyde and stained with phalloidin and DAPI. Fluorescence images were acquired using a Leica DMi8 microscope, and cellular fluorescence was quantified in ImageJ. For quantitative analysis by flow cytometry, HSFs and HDFs were harvested without fixation and analyzed on an Attune NxT flow cytometer (Thermo Fisher Scientific, USA). Data were gated on singlet and viable cells, and uptake was expressed as the percentage of FITC-positive cells.

For FAP knockdown experiments, HSFs were transfected with FAP-specific siRNA (forward sequence: 5′-GGUGGAUUCUUUGUUUCAACAtt-3′, reverse sequence: 5′-UGUUGAAACAAAGAAUCCACCtt-3′) or negative control siRNA (forward sequence: 5′-GGGAGUUAGGAUAUGUUAUGAtt-3′, reverse sequence: 5′-AUAACAUAUCCUAACUCCCCCtt-3′) (Suzhou GenePharma Co., Ltd., China) using Lipofectamine RNAiMAX (Thermo Fisher Scientific, USA) according to the manufacturer's instructions. After 48 h, cells were harvested for Western blot validation or subjected to uptake and viability assays as described above.

### 2.7 Cell viability

Cell viability of HSFs after treatment with free erastin and erastin-loaded sEV formulations was assessed using a Cell Counting Kit-8 (CCK-8; Beyotime, China). HSFs were seeded into 96-well plates and allowed to attach overnight. Cells were then treated with free erastin or sEV formulations at erastin-equivalent concentrations ranging from 0 to 80 μM for 24 h. At the end of treatment, CCK-8 solution was added to each well according to the manufacturer's instructions, and the plates were incubated at 37 °C for 1 h. The absorbance at 450 nm was measured using a microplate reader (Bio-Rad 680, USA).

### 2.8 5-Ethynyl-2′-deoxyuridine (EdU) proliferation assay

HSF proliferation following treatment was evaluated using an EdU incorporation assay (Beyotime, China). HSFs were seeded in 24-well plates and cultured overnight. Cells were then exposed to the indicated sEV formulations at an erastin-equivalent concentration of 15 μM for 12 h. After treatment, the culture medium was replaced with EdU working solution and cells were incubated for 5 h at 37 °C. Cells were then fixed with 4% paraformaldehyde for 15 min at room temperature and washed three times with PBS. Subsequent steps were performed according to the manufacturer's instructions. Fluorescence images were acquired using a Leica DMi8 inverted fluorescence microscope (Leica Microsystems, Germany), and the percentage of EdU-positive cells was quantified using ImageJ.

### 2.9 Wound healing assay

A wound healing assay was used to assess the effects of the different formulations on HSF migration. HSFs were seeded into 6-well plates and grown to approximately 90% confluence. A straight scratch was made in the cell monolayer using a sterile 1 mL pipette tip. Floating cells were removed by washing three times with PBS, and fresh medium containing the indicated treatments was added. Phase-contrast images of the scratch area were captured at 0, 12 and 24 h using an inverted microscope. The wound area at each time point was measured in ImageJ and normalized to the initial wound area. The migration rate was calculated as (A_0_ − A_n_)/A_0_ × 100%, where A_0_ is the wound area at 0 h and A_n_ is the wound area at the indicated time.

### 2.10 Transwell migration assay

HSF migration was further evaluated using a Transwell assay. Transwell-24 inserts with 8.0 μm-pore polycarbonate membranes (Solarbio, China) were placed into 24-well plates. Treated HSFs were resuspended in serum-free medium and seeded into the upper chambers, while medium containing 10% fetal bovine serum (FBS) was added to the lower chambers as a chemoattractant. After 24 h of incubation at 37 °C, cells on the upper surface of the membranes were gently removed with a cotton swab. Cells that had migrated to the lower surface were fixed, stained with crystal violet, and imaged under a bright-field microscope. The number of migrated cells was counted in randomly selected fields using ImageJ.

### 2.11 Western blot

For cell or tissue samples, western blotting was performed following a standard protocol as previously described [23]. The following primary antibodies were used: FAP (ab314456, Abcam, UK), type I collagen (COL I; 14695-1-AP, Proteintech, China), type III collagen (COL III; 22734-1-AP, Proteintech, China), α-smooth muscle actin (α-SMA; 67735-1-Ig, Proteintech, China), GPX4 (ab125066, Abcam, UK), ACSL4 (22401-1-AP, Proteintech, China), Transferrin Receptor (TRFC, A5865, Abclonol, China) and SLC7A11/xCT (26864-1-AP, Proteintech, China), 4-Hydroxynonenal (4-HNE, ab46545, Abcam, UK) as appropriate. GAPDH (10494-1-AP, Proteintech, China) or α-Tubulin (11224-1-AP, Proteintech, China) was used as loading controls. After washing with TBST, membranes were incubated with HRP-conjugated secondary antibodies for 1 h at room temperature. Protein bands were visualized using enhanced chemiluminescence and imaged on a chemiluminescence detection system.

### 2.12 Immunofluorescence analysis of fibrosis-related markers

Immunofluorescence staining was performed to visualize fibrosis-related marker expression in HSFs. Cells were cultured on coverslips in 24-well plates and treated with the indicated formulations for 24 h. After treatment, cells were fixed with 4% paraformaldehyde for 15 min at room temperature and washed with PBS, followed by permeabilization with 0.1% Triton X-100 for 10 min and blocking with 5% BSA for 1 h. Cells were then incubated overnight at 4 °C with primary antibodies against COL I, COL III and α-SMA, followed by incubation with appropriate fluorophore-conjugated secondary antibodies at room temperature in the dark. For dual-color collagen imaging, COL I and COL III were visualized with red and green fluorophores, respectively, and nuclei were counterstained with DAPI. Fluorescence images were acquired using a Leica DMi8 fluorescence microscope (Leica Microsystems, Germany).

### 2.13 Calcein-AM/propidium iodide (PI) cell cytotoxicity assay

Cell viability and death were assessed using a Calcein-AM/PI Cell Viability/Cytotoxicity Kit (Beyotime, China). HSFs were seeded in 24-well plates, allowed to attach overnight and treated with the indicated formulations at an erastin-equivalent concentration of 15 μM for 24 h. After treatment, cells were washed with PBS and incubated with Calcein-AM/PI working solution at 37 °C for 20 min in the dark according to the manufacturer's instructions. Stained cells were imaged directly using an inverted fluorescence microscope, with Calcein-AM-positive cells considered viable and PI-positive cells considered dead. Additionally, cells were analyzed on a CytoFLEX S flow cytometer (Beckman Coulter, USA; Calcein: FITC 525/40; PI: 610/20).

### 2.14 Lipid peroxidation and GSH assays

Lipid peroxidation and GSH depletion in HSFs and rabbit HS tissues were quantified using MDA and total GSH assay kits (Beyotime, China). Absorbance was measured with a microplate reader (SPARK 10M, TECAN, Switzerland), and MDA and GSH levels were calculated from standard curves and normalized to total protein content.

### 2.15 Flow cytometric analysis of total ROS and lipid ROS

ROS and lipid ROS were analyzed by flow cytometry. After the indicated treatments, HSFs were incubated with DCFH-DA (Beyotime, China) for total ROS or BODIPY™ 581/591C11 (Beyotime, China) for lipid ROS, following the manufacturers’ instructions. Cells were then washed, gently resuspended, and analyzed on an Attune NxT flow cytometer (Thermo Fisher Scientific, USA).

### 2.16 Mitochondrial membrane potential (JC-1) assay and ultrastructure

Mitochondrial membrane potential (Δψm) in HSFs was evaluated using a JC-1 assay kit (Dojindo, Japan). After treatment with the indicated formulations, cells were stained with JC-1 according to the manufacturer's protocol and analyzed by flow cytometry on an Attune NxT flow cytometer (Thermo Fisher Scientific, USA).

Mitochondrial ultrastructure was examined by transmission electron microscopy (TEM; Hitachi, Japan). After the indicated treatments, cells were fixed, dehydrated, embedded in resin and sectioned using standard procedures. Ultrathin sections were stained and observed under TEM to evaluate ferroptosis-related mitochondrial changes.

### 2.17 Labile Fe^2+^ imaging

Labile intracellular Fe^2+^ was visualized using FerroOrange (Dojindo, Japan). Treated HSFs were incubated with FerroOrange working solution according to the manufacturer's instructions, washed with PBS and imaged on a Leica DMi8 fluorescence microscope (Leica Microsystems, Germany). Fluorescence intensity was quantified in ImageJ from at least five randomly selected fields per sample.

### 2.18 Caspase-3 activity assay

Caspase-3 activity was measured using a caspase-3 activity assay kit (Beyotime, C1115) according to the manufacturer's instructions. Briefly, HSFs were treated with PBS, Er, sEVs^Er,^ or sEVs^ErF^ for 24 h. Cells were then lysed and incubated with the caspase-3 substrate (Ac-DEVD-pNA) for 2 h at 37 °C. Absorbance was measured at 405 nm using a microplate reader. Caspase-3 activity was normalized to the Control group.

### 2.19 Fabrication and characterization of sEVs^ErF^-loaded DMNPs

To enable localized, sustained intradermal delivery of sEVs^ErF^, DMNPs were fabricated using GelMA as the needle matrix and PVA as the backing layer. A photocrosslinkable GelMA solution (20% w/v) was prepared by dissolving solid GelMA in a 0.25% (w/v) LAP solution (2.5 mg/mL in PBS) at 40-50 °C for ∼15 min with intermittent gentle shaking until fully dissolved. sEVs^ErF^ were then mixed into the GelMA solution to obtain a final vesicle concentration of 4 × 10^11^ particles/mL. A sufficient volume of GelMA/sEVs^ErF^ mixture was added to fully fill the microneedle cavities of the PDMS mold, followed by vacuum degassing to remove trapped air. Excess GelMA/sEVs^ErF^ solution was removed from the mold surface to confine sEVs^ErF^ primarily within the needle tips. A 20% (w/v) PVA solution was then cast as the backing layer, followed by 405 nm photocrosslinking to crosslink the GelMA tip. The molds were then placed in a sealed chamber with silica gel desiccant at 4 °C until fully dried. Each patch contained 300 microneedles arranged in a circular array (diameter 1.2 cm) with an approximate tip diameter of 10 μm, base diameter of 300 μm, height of 700 μm and inter-needle spacing of 600 μm.

To determine actual sEV loading particle number in DMNPs, three randomly selected patches from different batches were dissolved in PBS, and particle numbers were quantified by NTA. The theoretical particle number per patch was calculated based on the microneedle tip volume and sEV concentration in GelMA. For batch-to-batch consistency analysis, erastin loading was measured in three independently prepared batches of sEVs^ErF^. The coefficient of variation (CV) was calculated as (SD/mean) × 100%.

### 2.20 Characterization of sEVs^ErF^-DMNPs

The overall geometry of DMNPs was documented using a digital camera, and surface morphology was examined by scanning electron microscopy (SEM; SU8600, Hitachi, Japan). Backing flexibility and conformability to irregular scar surfaces were evaluated by manually bending the patches and checking for cracking or delamination. For needle-cargo colocalization, 3,3′-dioctadecyloxacarbocyanine perchlorate (DiO)-labeled sEVs^ErF^ and red-labeled PVA backing were visualized on frozen DMNP sections by confocal microscopy to confirm that the fluorescent signal was confined to the microneedle region. The mechanical strength of DMNPs was assessed using a universal testing machine (Instron 5942, USA) in compression mode. Patches were placed on the platform with needle tips facing the upper probe and were compressed at 0.2 mm/min until visible deformation occurred, and the maximum load per patch was recorded.

### 2.21 Hemolysis assay

The hemolytic activity of sEVs^ErF^ and sEVs^ErF^-loaded dissolvable microneedle patches (sEVs^ErF^-DMNPs) was evaluated using a red blood cell (RBC) hemolysis assay. Fresh whole blood was collected from healthy rabbits into anticoagulant tubes and centrifuged to isolate RBCs. The RBC pellet was washed three times with sterile PBS until the supernatant was clear, and then resuspended in PBS to obtain a 2% (v/v) RBC suspension.

For testing, sEVs^ErF^ (in PBS) and sEVs^ErF^-DMNPs extracts (DMNP tips dissolved in PBS to yield an sEV-containing solution) were prepared at the indicated concentrations. PBS served as the negative control, and 1% (v/v) Triton X-100 served as the positive control. Equal volumes of each sample solution and the RBC suspension were mixed and incubated at 37 °C for 1 h with gentle shaking. After incubation, samples were centrifuged (e.g., 1000×*g*, 5 min) and the absorbance of the supernatant was measured at 540 nm using a microplate reader. Hemolysis (%) was calculated as:

$$\text{Hemolysis}\;\left(\%\right)=\left({\text{OD}}_{\text{sample}}-{\text{OD}}_{\text{PBS}}\right)\;/\;\left({\text{OD}}_{\text{Triton}}-{\text{OD}}_{\text{PBS}}\right)\times 100\%\text{.}$$

All measurements were performed in triplicate (n = 3 per group).

### 2.22 Establishment of rabbit ear HS model

After general anesthesia, four full-thickness circular dermal wounds (10 mm in diameter) were created on the ventral surface of each ear. The perichondrium on the wound bed was carefully removed, while the underlying cartilage was preserved intact. Mature HS tissues typically developed by day 28 after surgery. At this time point, rabbits were enrolled into subsequent treatment protocols. All animal procedures were approved by the Animal Welfare and Ethics Committee of Beijing Keyu Experimental Animal Breeding Center (approval code: KY20250310006) and conducted in accordance with institutional and national guidelines.

### 2.23 *In vivo* insertion and dissolution study of DMNPs

DMNPs (Ø 1.2 cm, 300 needles/patch) were applied to mature rabbit ear scars for 15-20 min to allow tip dissolution and then removed. The recovery of pinholes at the application sites was photographed with a digital camera at 0, 5, 10, 15 and 20 min to assess microchannel closure. Effective insertion was further verified by excising the ears immediately after DMNPs removal, staining the application sites with Trypan blue, gently rinsing with PBS and imaging the regularly arranged dark-blue pinholes. Microneedle dissolution was evaluated by measuring needle height before and after insertion. To compare intradermal retention between DMNP-mediated delivery and intradermal injection, sEVs^ErF^ were labeled with 1,1′-dioctadecyl-3,3,3′,3′-tetramethylindocarbocyanine perchlorate (DiI) and loaded into DMNPs as described in Section 2.19 or injected intradermally at an equivalent dose (2 × 10^9^ particles per site). Fluorescence signals at treated sites were monitored up to 7 days using an *in vivo* imaging system (IVIS, PerkinElmer, USA).

### 2.24 Animal experiment procedure

After confirmation of mature HS formation at day 28 post-surgery, rabbits were randomly divided into seven groups (n = 3 per group): normal skin (NS), HS control (HS), free erastin (Er), erastin-loaded sEVs (sEVs^Er^), FAP-targeted erastin-loaded sEVs (sEVs^ErF^), sEVs^ErF^-loaded DMNPs (sEVs^ErF^-DMNPs), and triamcinolone acetonide (TA) as a clinical reference treatment. For groups receiving erastin or sEV-based injections (Er, sEVs^Er^, sEVs^ErF^), formulations were administered intradermally into the scar tissue at a dose equivalent to 2.0 × 10^9^ particles per site for sEV-containing groups, with erastin concentration matched across groups. For the sEVs^ErF^-DMNPs group, one patch (Ø 1.2 cm, 300 needles) was applied per scar for 15-20 min to allow full dissolution of the microneedle tips, delivering an estimated payload of approximately 2.0 × 10^9^ particles per site based on casting volume. The HS group received an equal volume of PBS, and the TA group received intralesional TA at a clinically relevant dose.

Treatments were initiated once hypertrophic scars had fully formed (day 28 post-surgery) and were maintained until day 49. At the indicated endpoints, rabbits were euthanized and scar tissues, along with adjacent normal dermis when required, were collected for histological evaluation, biochemical assays, Western blotting and transcriptomic analysis as described in the corresponding sections. Systemic safety was evaluated at the terminal time point (day 49) following completion of the treatment course.

Rabbits were monitored daily throughout the treatment period for signs of pain, distress, or abnormal behavior, including changes in feeding, grooming, mobility, and vocalization. Application sites were visually inspected for erythema, edema, or other signs of irritation.

### 2.25 Histological analysis

For histological evaluation, scar tissues and adjacent normal skin (NS) were harvested at the indicated time points, fixed in 4% paraformaldehyde, dehydrated, embedded in paraffin and sectioned at 4-5 μm thickness. Hematoxylin and eosin (H&E) staining used standard histological protocols. Masson's trichrome staining was performed on adjacent sections to visualize collagen fibers. The scar elevation index (SEI) was calculated on H&E sections as the ratio of scar thickness to the thickness of adjacent normal dermis.

### 2.26 *In vitro* and i*n vivo* ferroptosis rescue experiments

HSFs were treated with sEVs^ErF^ (erastin-equivalent concentration: 15 μM) in the presence or absence of Ferrostatin-1 (Fer-1, 30 nM; MedChemExpress, USA). After 24 or 48 h, cells were processed for CCK-8 assay, α-SMA immunofluorescence, and C11-BODIPY 581/591 lipid ROS detection following the procedures described in Sections 2.7, 2.12, 2.15.

To further validate the role of ferroptosis *in vivo*, an additional group was included in which Fer-1 (50 μM in GelMA tips) was co-delivered with sEVs^ErF^-DMNPs. The treatment schedule was identical to the sEVs^ErF^-DMNPs group. Scar tissues were collected at day 49 for histological and western blot analysis.

### 2.27 Hematology and serum biochemistry

At day 49, blood samples were collected from all rabbits via ear vein puncture. Hematological parameters, including white blood cells (WBC), red blood cells (RBC), hemoglobin (HGB), hematocrit (HCT), mean corpuscular volume (MCV), and platelets (PLT), were analyzed using a Mindray 5180 CRP automated hematology analyzer (Shenzhen Mindray Bio-Medical Electronics Co., Ltd., China). Serum biochemical parameters, including alanine aminotransferase (ALT), aspartate aminotransferase (AST), alkaline phosphatase (ALP), urea, and creatinine, were measured using a Toshiba TBA120FR automatic biochemical analyzer (Toshiba, Japan) with reagents from Beijing Beijian-Xinchuangyuan Biotechnology Co., Ltd., China.

Serum IL-6 levels were quantified using a rabbit-specific IL-6 ELISA kit (MM-0302O1, Jiangsu Meimian) according to the manufacturer's instructions. Briefly, serum samples were added to pre-coated plates, incubated with detection antibody, and developed with TMB substrate. Absorbance was read at 450 nm using a microplate reader.

### 2.28 RNA sequencing and bioinformatic analysis

For bulk RNA sequencing, scar tissue from the wound center was collected at the terminal time point (day 49), immediately rinsed with RNase-free (DEPC-treated) water, and snap-frozen in liquid nitrogen. Total RNA extraction, library preparation (TIANSeq), Illumina paired-end 150 bp sequencing, and downstream bioinformatic analyses were performed by Tiangen Biotech (Beijing, China). Differentially expressed genes (DEGs) were identified using DESeq2 with thresholds of FDR <0.05 and |log_2_ fold change| ≥ 1 (i.e., ≥2-fold change). Gene Ontology (GO) and KEGG pathway enrichment analyses were conducted using default parameters, and enrichment p values were calculated accordingly.

### 2.29 Quantification and image analysis

For all imaging-based quantifications (cellular uptake, immunofluorescence, FerroOrange staining, and histological staining), a minimum of 5 randomly selected fields per sample were analyzed. All quantifications were performed in a blinded manner, with the analyst unaware of group allocation during image acquisition and analysis. For fluorescence-based quantifications, background fluorescence was subtracted using the mean intensity of regions without cells (for cellular imaging) or non-tissue areas (for histology). Fluorescence intensity values were normalized to the control group as indicated in the figure legends. For histological quantifications, background was subtracted using non-tissue areas, and consistent threshold settings were applied across all samples. ROIs were randomly selected from the entire field of view (cellular imaging) or from the dermal scar region (histology), with representative examples shown in Fig. S8A and S8B, respectively.

### 2.30 Statistical analysis

All quantitative data are presented as mean ± standard deviation (SD). Unless otherwise specified, each experiment was performed with at least three independent biological replicates. Statistical analyses were conducted using GraphPad Prism (version 9.0, GraphPad Software, USA). Differences between two groups were evaluated using an unpaired two-tailed Student's t-test. For comparisons among multiple groups, one-way analysis of variance (ANOVA) was applied, followed by appropriate post hoc tests. A p-value <0.05 was considered statistically significant.

## 3 Results

### 3.1 FAP upregulation in HSFs

**Fig. 1:**
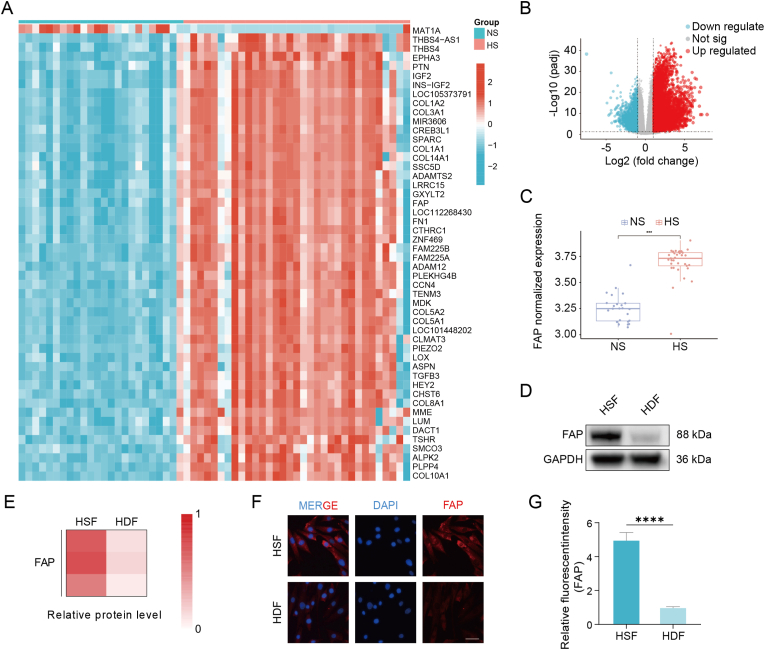
FAP is upregulated in HS tissues and fibroblasts. (A) Heatmap of top differentially expressed genes between HS (n = 33) and NS (n = 24), with FAP highlighted. (B) Volcano plot of DEGs showing marked upregulation of FAP. (C) Box plot of normalized FAP mRNA in NS vs HS. (D, E) Western blot and densitometric heatmap of FAP in HSFs and HDFs; GAPDH, loading control. (F) Immunofluorescence of FAP (red) in HSFs and HDFs; nuclei, DAPI (blue). Scale bar, 30 μm. (G) Quantification of FAP fluorescence (HSFs vs HDFs). Data are mean ± SD; ∗∗∗∗p < 0.0001.

FAP is a well-recognized biomarker of fibrotic disease [24]. To establish its relevance to HS, we integrated GSE178411 and GSE188952 with batch-effect correction and compared gene expression profiles between 33 HS and 24 normal skin (NS) samples. A total of 8,310 differentially expressed genes including FAP, were identified (|log_2_FC| > 1, p < 0.05) as shown in the heatmap and volcano plot (Fig. 1A and B). A box plot of the merged dataset demonstrated higher FAP mRNA expression in HS versus that in NS (Fig. 1C). Next, FAP protein expression in HSFs and HDFs was further confirmed by Western blotting. The results showed higher FAP in HSFs than that in HDFs (Fig. 1D and E). Consistently, the results of immunofluorescence demonstrated stronger FAP staining in HSFs than that in HDFs (Fig. 1F) with quantitative analysis confirming this difference (Fig. 1G). These findings validate FAP as a viable target for the treatment of HS and open the possibility for further use of FAP for anti-fibrotic approaches in other FAP-positive fibrotic diseases.

### 3.2 Preparation and characterization of erastin-loaded and FAP-targeted sEVs (sEVs^ErF^)

**Fig. 2:**
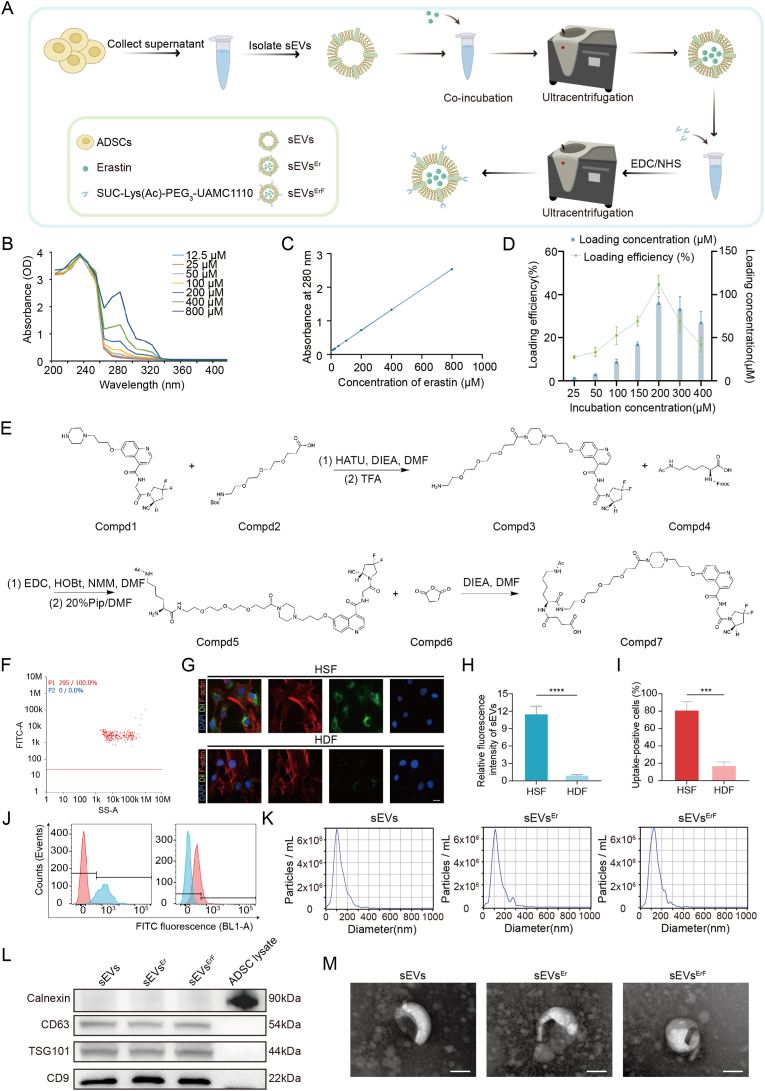
Construction and characterization of sEVs^ErF^. (A) Schematic of sEVs^ErF^ preparation from ADSC-derived sEVs, including erastin loading and UAMC1110 conjugation. (B, C) UV-Vis spectra of erastin and calibration curve at 280 nm. (D) Erastin loading content and loading efficiency at increasing incubation concentrations. (E) Synthetic route of SUC-Lys (Ac)-PEG_3_-UAMC1110. (F) Nano-flow cytometry of ligand-conjugated sEVs. (G) Fluorescence images of HSFs and HDFs incubated with SUC-Lys (FITC)-PEG_3_-UAMC1110-labeled sEVs^ErF^ (FITC, green; phalloidin, red; DAPI, blue). Scale bar, 10 μm. (H) Image-based quantification of cellular fluorescence. (I, J) Uptake-positive cells (%) and representative flow-cytometry histograms for HSFs (left) and HDFs (right). (K) NTA size distributions of sEVs, sEVs^Er^, and sEVs^ErF^. (L) Western blot analysis of sEV-positive markers (CD9, CD63, TSG101) and the negative marker Calnexin in parental ADSC lysates and the indicated sEV formulations (sEVs, sEVs^Er^, sEVs^ErF^). (M) TEM images showing cup-shaped morphology of the three vesicle types. Scale bar, 100 nm. Data are mean ± SD; ∗∗∗p < 0.001, ∗∗∗∗p < 0.0001.

We engineered erastin-loaded and FAP-targeted sEVs (sEVs^ErF^) via a modular workflow (Fig. 2A). sEVs isolated from ADSC-conditioned medium were co-incubated with erastin to obtain sEVs^Er^ and then ultracentrifuged to remove unencapsulated erastin. To quantify erastin loading with rigor, a full-wavelength UV-Vis absorption scan of erastin confirmed a maximal absorbance at 280 nm across 12.5-800 μM (Fig. 2B) and an absorbance-concentration calibration enabled subsequent loading quantification (Fig. 2C). This calibration underpinned all downstream loading analyses. The loading content and loading efficiency of erastin increased with increasing erastin incubation concentration, with a peak loading efficiency of approximately 47.9% at 200 μM (encapsulated concentration 89.59 ± 8.26 μM, CV = 9.22%) (Fig. 2D).

Subsequently, the surface engineering of sEVs^Er^ was achieved by EDC/NHS-mediated coupling of the synthesized ligand SUC-Lys (Ac)-PEG_3_-UAMC1110, followed by clearance of excess ligand by ultracentrifugation. The synthesis and architecture of SUC-Lys (Ac)-PEG_3_-UAMC1110 were detailed in Fig. 2E: UAMC1110, as the selective FAP-binding headgroup [17], a PEG_3_ linker to enhance solubility and mitigate steric hindrance [25], and a succinylated-acetyl-Lys handle enabling NHS-ester activation for efficient amide coupling to sEV surface amines [26]. The chemical identity and purity of SUC-Lys (Ac)-PEG_3_-UAMC1110 were confirmed by analytical HPLC (purity ∼99.78%) and MS (Fig. S1A and B). In parallel, an FITC-tagged analogue (SUC-Lys (FITC)-PEG_3_-UAMC1110) was synthesized for ligand tracking and quantitative labeling (Fig. S1C–E). A fluorescence standard curve (R^2^ = 0.9996) enabled estimation of ligand density on vesicles at ∼2.25 × 10^4^ ligands per sEV (Fig. S1F). Ligand density was estimated assuming spherical vesicles with uniform surface distribution. Nano-flow cytometry confirmed efficient ligand conjugation, evidenced by a FITC-positive vesicle population with a consistent fluorescence-intensity distribution (Fig. 2F).

Next, SUC-Lys (FITC)-PEG_3_-UAMC1110-labeled sEVs^ErF^ were incubated with HSFs and HDFs to test whether conjugation enhanced cellular selectivity. Fluorescence microscopy showed markedly stronger green signals in HSFs (Fig. 2G) and image-based quantification revealed ∼11-fold higher uptake of sEVs^ErF^ in HSFs versus that in HDFs (Fig. 2H). Consistently, flow cytometry confirmed preferential uptake at the population level (Fig. 2I), with bar-graph quantification in Fig. 2J. Beyond targeting outcomes, we evaluated the basic characteristics of sEVs^ErF^. Nanoparticle tracking analysis (NTA) showed a modest right-shift in size distribution while retaining a narrow nanoscale range (Fig. 2K). The expressions of landmark proteins on the surface of sEVs, sEVs^Er^, and sEVs^ErF^ were validated by Western blotting (CD9, CD63, TSG101 positive; calnexin negative) (Fig. 2L). Transmission electron microscopy (TEM) revealed the typical cup-shaped morphology for all three formulations, indicating preserved structural integrity throughout engineering (Fig. 2M). Together, these data confirm selective uptake of sEVs^ErF^ with maintained vesicle quality, motivating functional evaluation *in vitro*.

To further investigate the kinetics and specificity of FAP-mediated uptake, we performed time-course and competitive blocking experiments. As shown in Fig. S2A–B, the uptake of FITC-labeled sEVs^ErF^ by HSFs increased progressively from 0.5 to 12 h, with a gradual decrease in uptake rate after 6 h, suggesting saturation of FAP binding sites. Moreover, pre-incubation with excess free UAMC1110 significantly reduced cellular fluorescence by approximately 5- to 6-fold (Fig. S2C–D), confirming that the enhanced uptake is specifically mediated by FAP targeting.

To genetically validate FAP dependency, we knocked down FAP in HSFs using specific siRNA. Western blot analysis confirmed efficient FAP knockdown (Fig. S2E–F). FAP silencing significantly reduced the uptake of sEVs^ErF^ (Fig. S2G–H). Consistently, CCK-8 assays showed that FAP knockdown partially reversed sEVs^ErF^-induced inhibition of HSF viability at both 24 and 48 h (Fig. S2I), indicating that FAP is essential for both the selective uptake and the anti-fibrotic activity of sEVs^ErF^.

### 3.3 *In vitro* antifibrotic activity of sEVs^ErF^ in HSFs

**Fig. 3:**
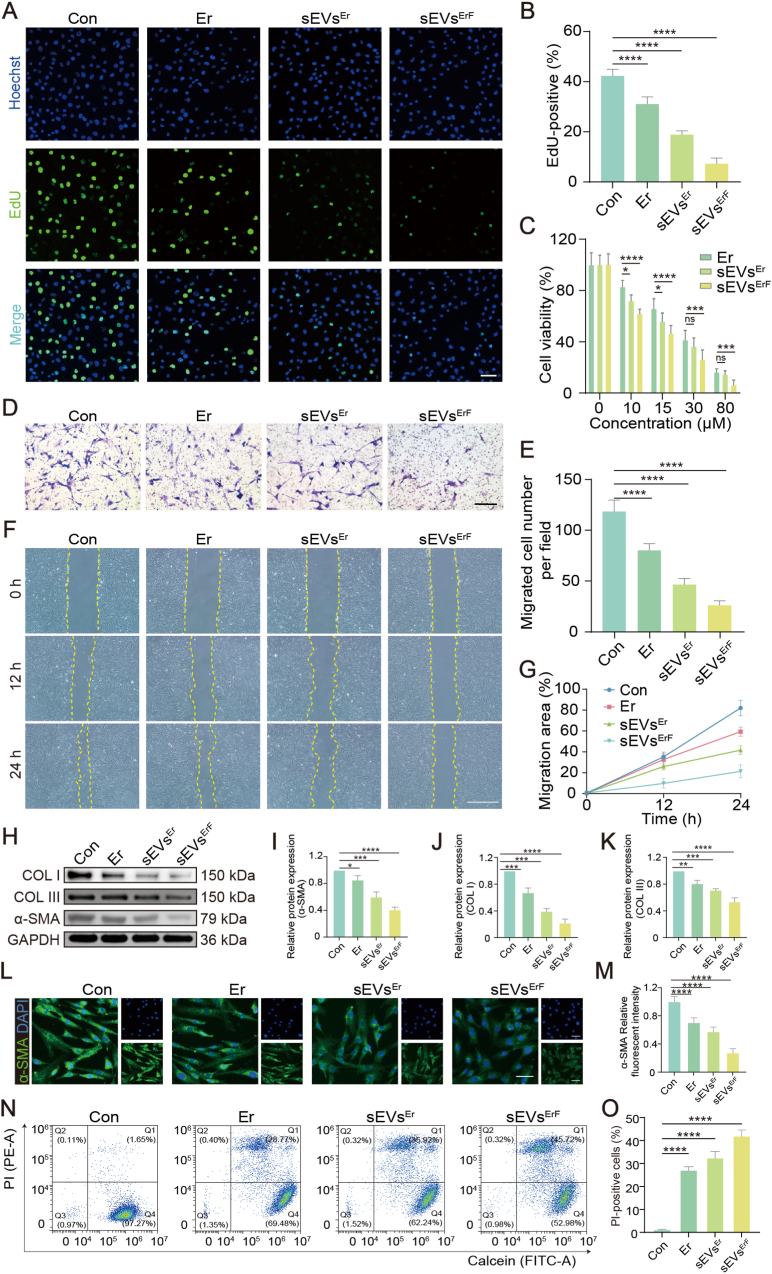
*In vitro* antifibrotic effects of sEVs^ErF^ on HSFs. (A, B) EdU staining and quantification of proliferating HSFs treated with Con, Er, sEVs^Er^, or sEVs^ErF^. Scale bar, 100 μm. (C) CCK-8 assay showing dose-dependent viability changes. (D, E) Transwell migration images and quantification of migrated HSFs. Scale bar, 100 μm. (F, G) Wound healing assay images and migration area (%) over time. Scale bar, 400 μm. (H-K) Western blot and densitometric analysis of COL I, COL III, and α-SMA; GAPDH, loading control. (L, M) α-SMA immunofluorescence and quantitative fluorescence intensity in HSFs. Scale bar, 50 μm. (N, O) Calcein-AM/PI flow cytometry and quantification of PI-positive cells. Data are mean ± SD; ∗p < 0.05, ∗∗p < 0.01, ∗∗∗p < 0.001, ∗∗∗∗p < 0.0001.

To determine the optimal treatment concentration of sEVs^ErF^, a CCK-8 assay was performed. As shown in Fig. 3C, all erastin-based formulations decreased HSF viability in a dose-dependent manner, with sEVs^ErF^ showing the strongest inhibitory effect. Accordingly, 15 μM, which produced approximately 50% inhibition of HSF viability, was selected for subsequent *in vitro* experiments.

We then evaluated the biological effects of sEVs^ErF^ on HSFs through a suite of *in vitro* assays assessing proliferation, viability, migration, and fibrosis-associated protein expression. As shown in Fig. 3A and B, EdU staining revealed a significant reduction in HSF proliferation after treatment with free erastin (Er), sEVs^Er^, or sEVs^ErF^, with the strongest inhibition observed for sEVs^ErF^. We then assessed the migration ability of HSFs using Transwell and wound-healing assays. Transwell analysis (Fig. 3D and E) showed that sEVs^ErF^ markedly reduced the number of migrated HSFs. Consistently, wound-closure assays (Fig. 3F and G) demonstrated slower healing rates and smaller closure areas in the sEVs^ErF^ group, indicating impaired migratory capacity. At the molecular level, we examined canonical fibrotic markers to determine whether these phenotypes were accompanied by matrix remodeling. Western blotting (Fig. 3H) showed that Er, sEVs^Er^, and especially sEVs^ErF^ substantially downregulated the expressions of COL I, COL III, and α-SMA, with quantification in Fig. 3I–K. These antifibrotic effects were further corroborated by α-SMA immunofluorescence (Fig. 3L). The results demonstrated that sEVs^ErF^-treated cells exhibited weaker α-SMA expression and quantitative analysis supported these observations (Fig. 3M). In parallel, immunofluorescence of collagen subtypes revealed a shift toward a more “repair-like” matrix: COL I (red) decreased relative to COL III (green), and the COL I/COL III fluorescence-intensity ratio declined progressively across groups (Control > Er > sEVs^Er^ > sEVs^ErF^) (Fig. S3A and B). Finally, Calcein-AM/PI staining and flow cytometry indicated that sEVs^ErF^ significantly increased the proportion of PI-positive cells (Fig. 3N and O), indicating severe damage to the cell membrane in the sEVs^ErF^ group. Because erastin canonically triggers ferroptosis, we hypothesized that the observed antifibrotic effects were probably mediated by ferroptotic signaling.

To dissect the contributions of the targeting ligand and erastin loading, we compared sEVs^F^ (UAMC1110-modified sEVs without erastin) with PBS, unmodified sEVs, and sEVs^ErF^ (Fig. S3). α-SMA immunofluorescence showed a moderate reduction in sEVs^F^-treated HSFs compared to controls (Fig. S3C and D). CCK-8 assays revealed that sEVs^F^ also inhibited cell viability, with effects reaching significance by 48 h (Fig. S3E). In contrast, sEVs^ErF^ treatment resulted in markedly stronger inhibition of both α-SMA expression and cell viability at all time points, significantly outperforming sEVs^F^ (p < 0.001). These results indicate that while UAMC1110 targeting alone confers a moderate anti-fibrotic effect, the combination with erastin loading is required for maximal efficacy.

### 3.4 Ferroptosis as a mechanism underlying the antifibrotic activity of sEVs^ErF^

To validate whether the antifibrotic effects of sEVs^ErF^ on HSFs were mediated by ferroptosis, we performed biochemical, morphological, and molecular assays targeting hallmark features of this pathway, including lipid peroxidation, GSH depletion, iron accumulation, mitochondrial dysfunction, and dysregulation of ferroptosis-related proteins.

**Fig. 4:**
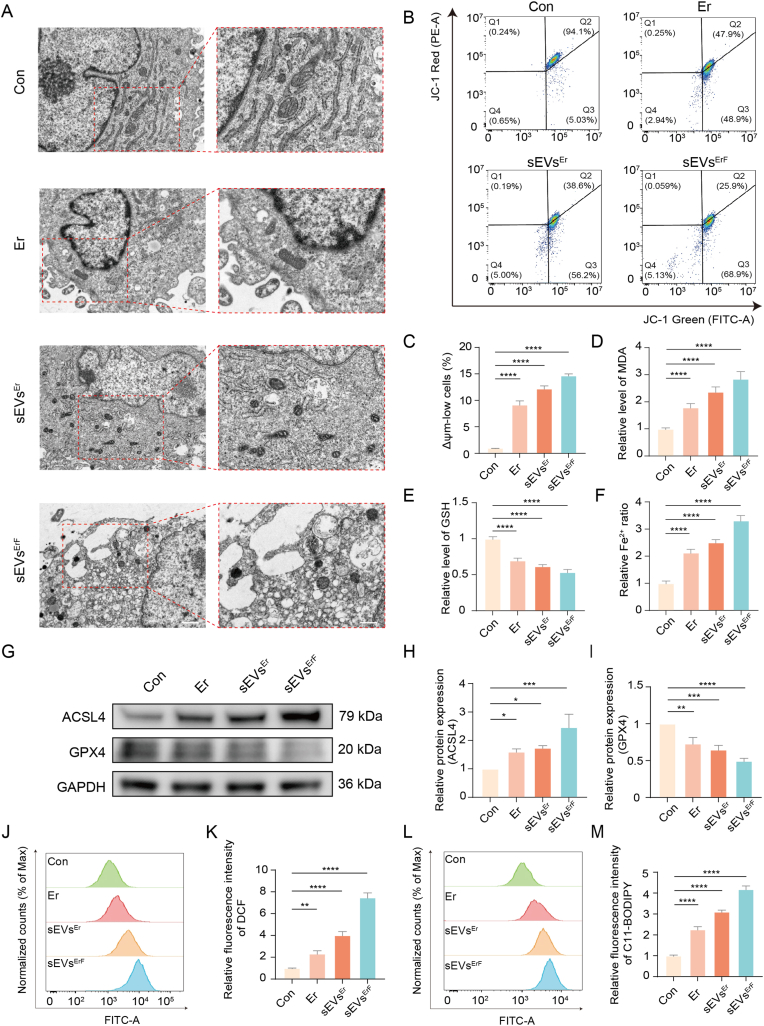
sEVs^ErF^ induces ferroptosis in HSFs. (A) TEM images of mitochondria in HSFs after the indicated treatments, showing ferroptosis-like damage in the sEVs^ErF^ group (reduced volume, dense membranes, loss of cristae). Scale bars, 1 μm (left) and 500 nm (right). (B, C) Representative flow-cytometry plots of JC-1 and percentage of Δψm-low cells. (D, E) Cellular MDA and GSH levels. (F) Labile Fe^2+^ content measured by FerroOrange. (G-I) Western blots of ACSL4 and GPX4 and corresponding densitometric analysis. (J, K) Total ROS assessed by DCFH-DA flow cytometry and quantitative fluorescence. (L, M) Lipid ROS measured by C11-BODIPY 581/591 and corresponding quantification. Data are mean ± SD; ∗p < 0.05, ∗∗p < 0.01, ∗∗∗p < 0.001, ∗∗∗∗p < 0.0001.

First, we examined ultrastructure by TEM. sEVs^ErF^-treated cells displayed typical ferroptotic mitochondrial damage, including reduced mitochondrial volume, increased membrane density, and diminished cristae (Fig. 4A). Concordantly, mitochondrial membrane potential measurements indicated functional impairment. Loss of mitochondrial membrane potential (Δψm) was further evidenced by JC-1 staining. The results showed that sEVs^ErF^ shifted fluorescence from red aggregates to green monomers (Fig. 4B) and quantification was performed as the percentage of Δψm-low cells (JC-1 monomer-high/aggregate-low population) (Fig. 4C).

As a terminal product of lipid peroxidation, malondialdehyde (MDA) was markedly increased by Er, sEVs^Er^, and most prominently by sEVs^ErF^ (Fig. 4D) [27]. In parallel, intracellular GSH, a critical antioxidant that limits ferroptosis by reducing lipid hydroperoxides via GPX4, was significantly depleted after sEVs^ErF^ treatment (Fig. 4E) [28]. To mechanistically anchor these redox shifts to iron-dependent chemistry, we quantified labile Fe^2+^. Labile iron accumulation, a driver of Fenton chemistry and lipid reactive oxygen species (ROS) during ferroptosis [29], was visualized with the FerroOrange probe (representative images in Fig. S4A) and quantification confirmed elevated Fe^2+^ levels in the sEVs^ErF^ group (Fig. 4F).

To connect the above phenotypes with ferroptosis-related proteins, the expression levels of acyl-CoA synthetase long-chain family member 4 (ACSL4) and GPX4 were detected. The results demonstrated the upregulation of ACSL4, which facilitated polyunsaturated acyl-chain enrichment and lipid peroxidation, and downregulation of GPX4 as a central lipid hydroperoxide reductase (Fig. 4G–I) [30]. In addition, flow cytometry revealed a significant increase in cellular ROS following sEVs^ErF^ exposure (Fig. 4J and K). Importantly, this increase extended to lipid peroxides which are a defining feature of ferroptosis. Lipid ROS measured by C11-BODIPY flow cytometry were increased in the sEVs^ErF^ group (Fig. 4L and M), corroborating enhanced lipid peroxidation [28].

To further confirm that these ferroptotic features directly underlie the anti-fibrotic effects, we performed rescue experiments using the ferroptosis inhibitor Fer-1. Functionally, co-treatment with Fer-1 significantly reversed sEVs^ErF^-induced reductions in HSF viability, with marked rescue effects observed at both 24 and 48 h (Fig. S4B). At the phenotypic level, Fer-1 partially restored α-SMA expression levels, indicating that the loss of myofibroblast identity was at least partly ferroptosis-dependent (Fig. S4C and D). Most importantly, as lipid peroxidation is the terminal execution step of ferroptosis, we examined whether Fer-1 could block this key event. C11-BODIPY staining showed that sEVs^ErF^-induced lipid peroxidation was abolished by Fer-1, with no significant difference between Control and sEVs^ErF^ + Fer-1 groups (Fig. S4E and F). Together, these rescue experiments confirm that the anti-fibrotic effects of sEVs^ErF^ are specifically mediated by ferroptosis.

To further differentiate ferroptosis from other cell death modalities, we assessed caspase-3 activity as a marker of apoptosis. As shown in Fig. S4G, no significant differences in caspase-3 activity were observed among Control, Er, sEVs^Er^, and sEVs^ErF^ groups, indicating that apoptosis is not a major contributor to the cell death induced by our formulations. Having established a ferroptotic mechanism *in vitro*, we next addressed the delivery barrier *in vivo* by employing dissolvable microneedles to achieve localized intradermal delivery.

### 3.5 Transdermal delivery of sEVs^ErF^ via dissolvable microneedle patches

**Fig. 5:**
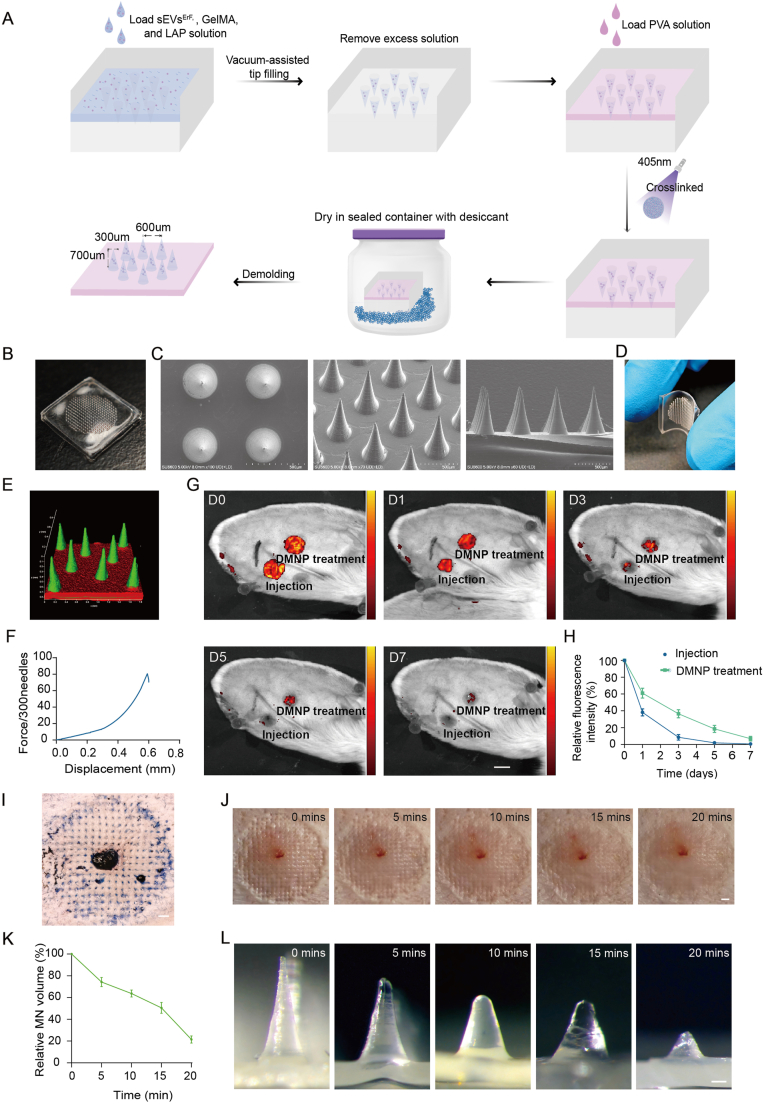
Characterization and performance of sEVs^ErF^-loaded dissolvable microneedle patches (sEVs^ErF^-DMNPs). (A) Schematic of GelMA-PVA bilayer microneedle fabrication and loading of sEVs^ErF^ into needle tips. (B) Photograph of the microneedle array. (C) SEM images showing microneedle geometry from different views. Scale bar, 500 μm. (D) Bending test of the PVA backing demonstrating flexibility. (E) Confocal 3D reconstruction of DiO-labeled sEVs^ErF^ (green) localized in GelMA tips and red-labeled PVA backing. (F) Compression test showing mechanical strength of the patch. (G, H) *In vivo* fluorescence imaging of DiI-labeled sEVs^ErF^ delivered by DMNP or intradermal injection and corresponding signal decay over 7 days. Scale bar, 1 cm. (I) Trypan-blue staining of rabbit ear skin showing microchannels created by microneedles. Insertion success rate: 88.3 ± 2.9% (n = 3 patches, 300 needles per patch). Scale bar, 1 mm. (J) Closure of puncture sites within 20 min after patch removal. Scale bar, 1 mm. (K, L) Time-dependent reduction in microneedle height and representative images during dissolution. Scale bar, 100 μm. Data are mean ± SD.

Transdermal access for sEVs^ErF^ delivery is hindered by the stratum corneum [31]. To achieve localized and sustained delivery of sEVs^ErF^ to HS, we constructed sEVs^ErF^-loaded DMNPs (sEVs^ErF^-DMNPs). The needle tips were fabricated with 20% (w/v) GelMA and sEVs^ErF^, while the backing layer consisted of flexible 20% (w/v) PVA (Fig. 5A). Fabrication involved sequential casting of GelMA and PVA into a polydimethylsiloxane (PDMS) mold with 700 μm conical cavities, followed by 405 nm photopolymerization to ensure tip crosslinking and robust interlayer adhesion.

First, we verified geometric fidelity and surface sharpness of the array. The resulting patch displayed a uniform conical array with intact structural integrity (Fig. 5B). Consistently, SEM from vertical, oblique, and horizontal views confirmed sharp and consistent tips (Fig. 5C). Next, because conformal skin contact is critical in scars with irregular topography, we assessed backing compliance. Manual bending tests showed that the PVA backing tolerated deformation without cracking, indicating good conformability to irregular scar surfaces (Fig. 5D). Confocal imaging of DiO-labeled sEVs^ErF^-DMNPs showed intense green fluorescence confined to the microneedle tips, whereas the PVA backing was visualized by a red fluorophore, confirming successful fabrication of the GelMA/PVA bilayer microneedles and localization of the sEVs^ErF^ payload within the tip region (Fig. 5E). Compression testing indicated that each patch withstood a maximum load of ∼81 N (Fig. 5F). Given 300 needles per patch, this corresponds to ∼0.27 N per needle on average (81/300 N), which falls within the commonly reported insertion-force range for microneedles (0.1-0.5 N per needle) [32].

For DMNPs, based on the microneedle geometry (300 conical needles per patch, height 700 μm, base diameter 300 μm), the total tip volume was calculated as approximately 4.95 μL, corresponding to a theoretical loading of ∼2.0 × 10^9^ particles per patch at a sEV concentration of 4.0 × 10^11^ particles/mL in GelMA. Quantitative analysis by NTA revealed an actual loading of 1.80 ± 0.171 × 10^9^ particles per patch from three independent batches, with an inter-batch CV of 9.5%.

Functionally, we then compared depot behavior *in vivo*. DiI-labeled sEVs^ErF^ were delivered via the DMNPs or by intradermal injection at an equivalent dose (2 × 10^9^ particles per site). Fluorescence imaging revealed sustained signal in the DMNPs group over 7 days, whereas injection-group signals declined rapidly and were nearly undetectable by day 5 (Fig. 5G). Quantification confirmed significantly prolonged retention of sEVs^ErF^ delivered by DMNPs, demonstrating a depot effect (Fig. 5H). Further comparison of the decay kinetics showed that DMNP delivery extended the local retention of sEVs^ErF^ compared to intradermal injection, as reflected by a prolonged residence half-life and an approximately 2-fold increase in area under the curve (AUC0-7d) (Table S3). These data confirm that DMNP-mediated delivery achieves sustained local retention and prolonged exposure.

To confirm barrier traversal, we visualized microchannels at the application site in ex vivo rabbit ear skin using Trypan blue (Fig. 5I). Puncture marks closed rapidly within approximately 20 min (Fig. 5J), indicating rapid recovery of the microchannels created by microneedle insertion. Needle dissolution showed a time-dependent reduction in needle height (Fig. 5K), with representative images shown in Fig. 5L. Additionally, hemolysis assays showed minimal hemolysis for both sEVs^ErF^ and sEVs^ErF^-DMNPs (Fig. S5A and B), indicating no obvious hemolytic effect under the tested conditions. Encouraged by sustained intradermal retention and these preliminary safety-relevant assessments, we proceeded to therapeutic testing in a rabbit HS model.

### 3.6 Therapeutic efficacy of sEVs^ErF^-DMNPs in a rabbit ear hypertrophic scar model

**Fig. 6:**
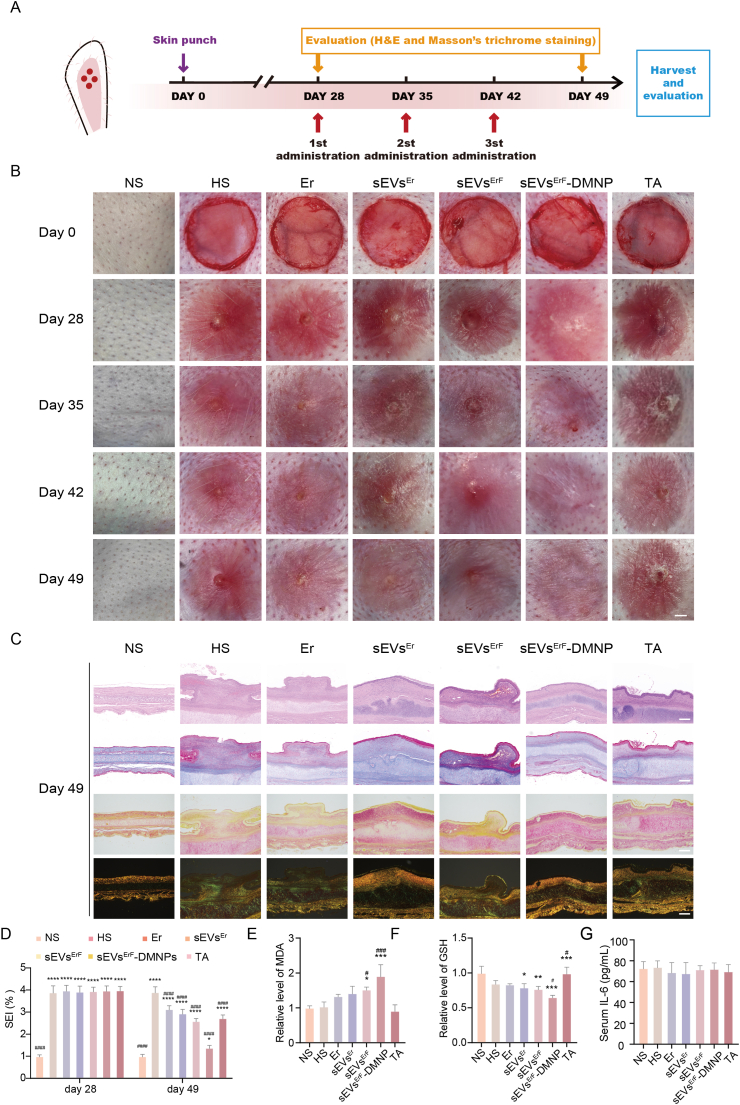
*In vivo* anti-scar efficacy of sEVs^ErF^-DMNPs in a rabbit HS model. (A) Experimental scheme of rabbit ear HS induction and treatment schedule. (B) Macroscopic images of scars in each group at day 0, 28, 35, 42, and 49, showing flatter and lighter scars in the sEVs^ErF^-DMNPs group. Scale bar, 2 mm. (C) Day-49 histology of scar sites: H&E, Masson's trichrome, and Sirius Red (bright-field and polarized light), demonstrating reduced dense collagen bundles and a shift toward type III collagen in the sEVs^ErF^-DMNPs group. Scale bars, 400 μm. (D) Scar elevation index (SEI) at day 28 and day 49. (E, F) MDA and GSH levels in scar tissues of the seven groups. (G) Serum IL-6 levels in the seven treatment groups at day 49 (n = 3). Data are mean ± SD; ∗p < 0.05, ∗∗p < 0.01, ∗∗∗p < 0.001, ∗∗∗∗p < 0.0001 vs NS; #p < 0.05, ##p < 0.01, ###p < 0.001, ####p < 0.0001 vs HS. n = 3 rabbits per group.

To investigate *in vivo* therapeutic efficacy of sEVs^ErF^ and, in particular, the DMNP format, we employed a rabbit ear HS model. Specifically, four full-thickness circular wounds (1 cm diameter) were created on the ventral surface of each ear with perichondrium removed to promote excessive fibroproliferation (Fig. 6A), reliably inducing raised and collagen-rich HS by postoperative day 28 [33]. At this time point, treatment was initiated as per the predefined schedule with three once-weekly doses (Fig. 6B). Seven groups were included: NS (untreated normal skin; reference control), untreated HS, and HS treated with Er, sEVs^Er^, sEVs^ErF^, sEVs^ErF^-DMNPs, and triamcinolone acetonide (TA), a clinically used anti-scar agent [34].

We first examined histological endpoints to capture overall scar architecture. Baseline histology at day 28 (before the first-treatment) confirmed the hypertrophic phenotype with thickened dermis and densely packed and disorganized collagen by hematoxylin and eosin (H&E) and Masson's staining (Fig. S5A). After three weekly treatments (day 49), the sEVs^ErF^-DMNPs group showed the most pronounced improvement on multi-modal histology (Fig. 6C): H&E revealed a thinner dermis with more regularly aligned fibroblasts approaching NS; Masson's trichrome showed reduced dense collagen bundles and improved fiber alignment; Sirius Red under white/polarized light demonstrated a shift toward finer and green-yellow type III collagen with relative reduction of thick orange-red type I fibers, indicative of more balanced matrix remodeling [35]. Consistent with morphological recovery, the scar elevation index (SEI) declined significantly from day 28 to day 49 in the sEVs^ErF^-DMNPs group, outperforming all comparators including TA (Fig. 6D). To link morphology to mechanism, we profiled biochemical markers of ferroptosis in scar tissue. Biochemical readouts within the scar further supported on-target ferroptotic activity: MDA was highest in the sEVs^ErF^-DMNPs group among the seven cohorts (Fig. 6E), whereas GSH was lowest (Fig. 6F), aligning with enhanced lipid peroxidation and GSH consumption observed *in vitro*.

Given the potential clinical translation of this platform, we next systematically assessed its systemic and local safety. As summarized in Tables S1 and S2, no statistically significant differences were observed across any of the seven treatment groups for all hematological parameters (WBC, RBC, HGB, HCT, MCV, PLT) and serum biochemical markers (ALT, AST, ALP, urea, creatinine) tested (p > 0.05). Serum IL-6 levels were also comparable across all groups (Fig. 6G). Throughout the treatment period, no adverse behavioral changes or signs of local irritation (e.g., erythema, edema) were observed in any group. Consistent with these findings, H&E staining of major organs (heart, liver, spleen, lung, and kidney) revealed no overt histopathological abnormalities across groups (Fig. S5B). Collectively, these data demonstrate the favorable systemic and local safety profile of our platform.

### 3.7 Molecular and transcriptomic validation of antifibrotic and ferroptosis-related responses *in vivo*

**Fig. 7:**
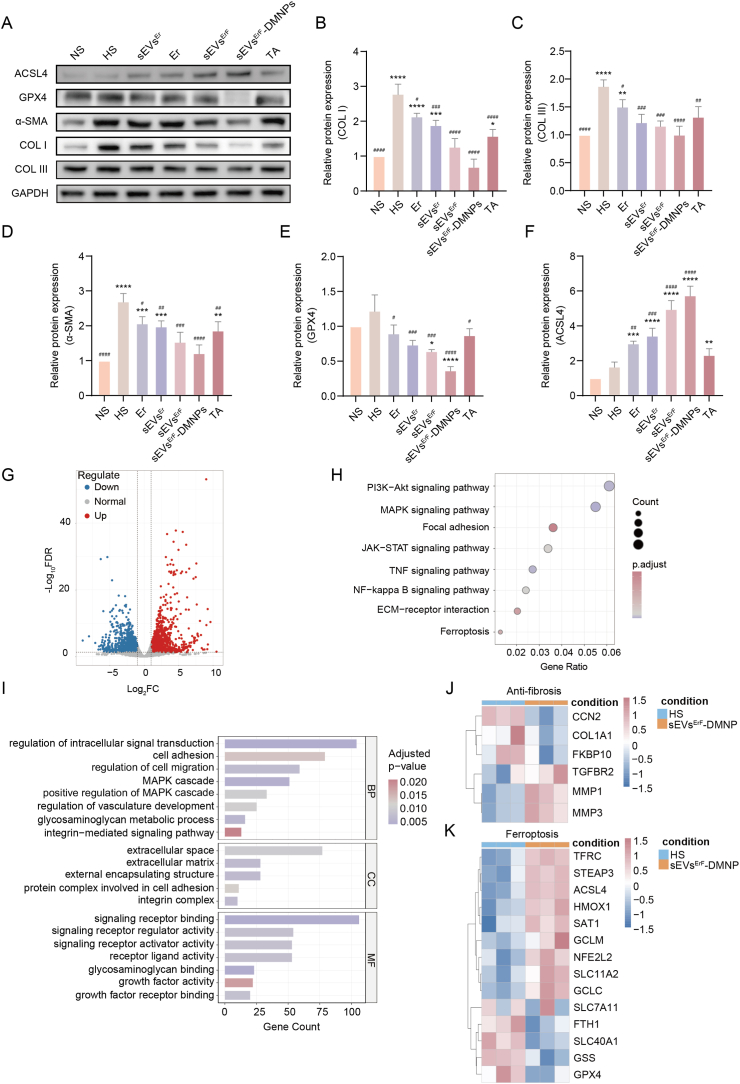
*In vivo* molecular validation of antifibrotic and ferroptosis-related changes. (A) Western blots of COL I, COL III, α-SMA, GPX4, and ACSL4 in scar tissues from NS, HS, Er, sEVs^Er^, sEVs^ErF^, sEVs^ErF^-DMNPs, and TA groups; GAPDH, loading control. (B-D) Densitometric analysis of COL I, COL III, and α-SMA relative to HS. (E, F) Densitometric analysis of GPX4 and ACSL4 relative to HS. (G) Volcano plot of DEGs between HS and sEVs^ErF^-DMNPs tissues (RNA-seq). (H) KEGG enrichment bubble plot showing fibrosis- and ferroptosis-related pathways. (I) GO enrichment (BP, CC, MF) highlighting ECM remodeling, glycosaminoglycan metabolism, and stress signaling. (J) Heatmap of fibrosis-related genes (e.g., CCN2, COL1A1, FKBP10, TGFBR2, MMP1, MMP3). (K) Heatmap of ferroptosis-related genes, including ACSL4, STEAP3, TFRC, SLC11A2, GPX4, GSS, FTH1, SLC40A1, NFE2L2, GCLM, GCLC, HMOX1, and SLC7A11/xCT, in scar tissues from HS and sEVs^ErF^-DMNPs groups. Data are mean ± SD; ∗p < 0.05, ∗∗p < 0.01, ∗∗∗p < 0.001, ∗∗∗∗p < 0.0001 vs NS; #p < 0.05, ##p < 0.01, ###p < 0.001, ####p < 0.0001 vs HS.

To mechanistically link the therapeutic effects observed *in vivo* to underlying pathways, we profiled fibrosis- and ferroptosis-related markers in rabbit scar tissues across seven groups (NS, HS, Er, sEVs^Er^, sEVs^ErF^, sEVs^ErF^-DMNPs, TA). Western blotting showed that the fibrotic proteins such as COL I, COL III, and α-SMA were all reduced by the above treatments with sEVs^ErF^-DMNPs producing the most pronounced decrease relative to untreated HS (Fig. 7A–D) [36]. Ferroptosis-related proteins were also modulated: sEVs^ErF^-DMNPs decreased GPX4 and increased ACSL4 most strongly among groups (Fig. 7A, quantified in Fig. 7E and F), consistent with the *in vitro* ferroptotic trends [37,38].

To establish causal relevance *in vivo*, an additional rescue cohort was included in which Fer-1 was co-delivered with sEVs^ErF^-DMNPs (Fig. S7). Histological analysis showed that Fer-1 co-treatment partially reversed the sEVs^ErF^-DMNPs-induced reduction in scar elevation index (Fig. S7A–B). Western blot analysis revealed that Fer-1 co-treatment significantly restored the expression of fibrotic markers (COL I, COL III, α-SMA) and ferroptosis-related proteins (GPX4, ACSL4, xCT, TFRC) (Fig. S7C–K). These *in vivo* rescue data further demonstrate that the therapeutic efficacy of sEVs^ErF^-DMNPs is specifically mediated by ferroptosis.

At the transcriptome scale, bulk RNA-seq comparing HS vs sEVs^ErF^-DMNPs revealed a broad transcriptional response. Differentially expressed genes (DEGs) were defined as having a minimum of two-fold change in expression relative to the untreated HS group (Fig. 7G). A total of 3394 DEGs were identfied, including 1665 upregulated genes and 1729 downregulated genes. In the Gene Ontology (GO) analysis, the most significantly enriched terms were clustered in fibrosis-related processes, including extracellular matrix (ECM) remodeling and glycosaminoglycan metabolism, and in ferroptosis-relevant stress-responsive processes involving the MAPK cascade (Fig. 7I). Kyoto Encyclopedia of Genes and Genomes (KEGG) enrichment highlighted multiple fibrosis-related pathways, including PI3K/Akt, MAPK, JAK/STAT, TNF, NF-κB, focal adhesion and ECM-receptor interaction. In addition, DEGs were enriched in the ferroptosis pathway, consistent with a ferroptosis-based anti-fibrotic mechanism of sEVs^ErF^-DMNPs (Fig. 7H).

To visualize module-level coherence, gene-set heatmaps showed coordinated modulation within fibrosis and ferroptosis panels. An anti-fibrosis panel showed down-modulation of CCN2 (CTGF), COL1A1, FKBP10, and TGFBR2, together with increases in MMP1/MMP3, indicating suppressed collagen synthesis/processing and enhanced remodeling (Fig. 7J) [39]. A ferroptosis panel demonstrated coordinated changes in ACSL4, STEAP3, TFRC, SLC11A2, GPX4, GSS, FTH1, and SLC40A1 (Fig. 7K). Within the NRF2-GSH metabolic axis, NFE2L2 and GCLM were significantly upregulated, while GCLC and SLC7A11/xCT showed no significant changes at the transcript level [40]. Notably, the lack of significant transcriptional changes in certain genes does not preclude functional pathway engagement, as ferroptosis is primarily regulated at the protein level, consistent with our protein-level findings (GPX4, ACSL4, xCT, TFRC, and 4-HNE; Fig. S7).

## 4 Discussion

HS is a common fibrotic outcome following skin injury, pathologically defined by excessive deposition of ECM [1]. HSFs are the central drivers of this process, exhibiting sustained proliferation, migration, and myofibroblast differentiation [41]. Current clinical interventions, such as corticosteroid injections, surgical excision, and laser therapy, often yield only transient symptomatic relief and are substantially limited by high recurrence rates [2]. In this study, we developed an integrated and precision-targeted therapeutic strategy that synergistically combines three core components: selective cellular recognition via FAP, ferroptosis-driven eradication of HSFs, and sustained local drug delivery enabled by DMNPs. This modular platform demonstrated selective accumulation in HSFs, effective activation of the SLC7A11/GSH/GPX4 ferroptosis signaling axis, and significant inhibition of collagen overproduction both *in vitro* and *in vivo*.

Achieving targeted delivery remains a fundamental prerequisite for precision anti-fibrotic therapy. To support reproducible vesicle engineering and to follow widely used EV isolation practices, we isolated sEVs by differential ultracentrifugation and validated vesicle identity and integrity using orthogonal assays (TEM, NTA, EV markers, and a negative marker) prior to cargo loading and surface conjugation [42]. Among potential molecular targets, FAP is predominantly expressed in fibroblasts and is highly upregulated in HSFs and other fibrotic microenvironments, rendering it an ideal target [16]. Notably, even if trace expression exists in non-fibroblast populations, clinical FAP-imaging studies have demonstrated extremely low background signal in healthy tissues with specific enrichment in fibrotic lesions [43], supporting the feasibility of FAP-directed targeting. Although prior studies have explored antibody- or peptide-based targeting strategies, such as integrin α5-binding peptide-decorated EVs for cancer-associated fibroblasts [44] and single-chain anti-FAP antibody-modified EVs for cardiac myofibroblasts [45], these approaches face practical challenges including complex genetic manipulation steps, conformational constraints, potential immunogenicity, and difficulties in reproducible manufacturing. In contrast, the small-molecule FAP inhibitor UAMC1110 offers high binding affinity, specificity, and suitability for controlled chemical conjugation [17]. By covalently grafting UAMC1110 onto sEV membranes via EDC/NHS-mediated amide coupling, we achieved a chemically defined surface presentation of the small-molecule ligand. This approach provided a robust and scalable route for sEV surface engineering while preserving vesicle integrity. Functionally, the engineered sEVs exhibited significantly enhanced cellular uptake in HSFs over HDFs, leading to potent inhibition of profibrotic activities and marked downregulation of α-SMA, COL I, and COL III expression. The FAP-specific uptake was further corroborated by competitive blocking experiments, where free UAMC1110 markedly inhibited sEVs^ErF^ internalization, and by genetic knockdown of FAP, which significantly reduced both uptake and anti-fibrotic efficacy. These results collectively confirm that the enhanced uptake and therapeutic activity of sEVs^ErF^ are strictly dependent on FAP expression. Of note, experiments using UAMC1110-modified sEVs without erastin loading (sEVs^F^) revealed that the targeting ligand itself exerts a moderate inhibitory effect on HSFs, consistent with its known function as a FAP enzyme inhibitor [17].

Ferroptosis has recently emerged as an attractive strategy for antifibrotic therapy. In fibrotic tissues, activated fibroblasts undergo metabolic reprogramming, characterized by iron overload, polyunsaturated lipid enrichment, and weakened antioxidant defense, which renders them intrinsically more vulnerable to ferroptosis than quiescent fibroblasts [8,9]. This iron-dependent form of regulated cell death is characterized by GSH depletion, lethal lipid peroxidation, and mitochondrial damage, and has been implicated in a range of conditions including cancer [46], neurodegenerative disorders [47], and organ fibrosis [48]. Among known ferroptosis inducers, erastin acts as a canonical small-molecule inhibitor of the cystine/glutamate antiporter system Xc^−^, leading to intracellular GSH depletion and subsequent inactivation of GPX4 [10]. However, the translational development of erastin has been hampered by its poor aqueous solubility and dose-limiting off-target toxicity [11]. By encapsulating erastin within FAP-directed sEVs, we enabled selective intracellular drug release specifically in HSFs, thereby minimizing exposure to healthy cells.

Treatment with sEVs^ErF^ triggered a comprehensive ferroptotic response, evidenced by elevated MDA, reduced GSH levels, intracellular Fe^2+^ accumulation, mitochondrial membrane depolarization, ACSL4 upregulation, and GPX4 downregulation. To establish causality, we performed rescue experiments using the ferroptosis inhibitor Fer-1. In vitro, co-treatment with Fer-1 significantly reversed sEVs^ErF^-induced reductions in HSF viability and α-SMA expression, and fully reversed lipid peroxidation. In vivo, co-delivery of Fer-1 with sEVs^ErF^-DMNPs substantially attenuated the therapeutic effects, as evidenced by increased SEI and restoration of fibrotic and ferroptosis-related protein expression. Mechanistically, ferroptosis is distinct from other cell death modalities such as apoptosis, which is characterized by caspase activation and nuclear fragmentation. In our study, the absence of caspase-3 activation excludes apoptosis as a primary mechanism. Instead, the hallmark ferroptosis features, including iron-dependent lipid peroxidation, GSH depletion, mitochondrial membrane depolarization, and characteristic mitochondrial ultrastructure—collectively support that sEVs^ErF^ activates the canonical “erastin-GSH-GPX4” pathway. While some genes within the NRF2-GSH metabolic axis did not show significant changes at the transcript level, this is not unexpected given that ferroptosis regulation involves complex post-transcriptional and post-translational mechanisms. Indeed, GPX4 activity is primarily regulated at the protein level, and our data clearly demonstrate decreased GPX4 protein expression alongside increased lipid peroxidation markers. Together, these findings confirm that the anti-fibrotic effects of sEVs^ErF^ are specifically mediated by ferroptosis, rather than other cell death pathways.

The dense nature of HS tissue presents a major barrier to effective drug penetration and retention, underscoring the need for advanced local delivery strategies [49]. To address this, we incorporated sEVs^ErF^ into DMNPs designed for painless, minimally invasive, and spatially precise intradermal administration [50,51]. The DMNPs, composed of GelMA-based needle tips and a flexible PVA backing layer, exhibited sufficient mechanical strength for reliable skin insertion, rapid dissolution within 20 min, and sustained intradermal retention for over seven days. This configuration enabled controlled release of therapeutic sEVs within the dermal layer, maintaining effective local drug concentrations over an extended period. Compared to conventional intradermal injection, DMNP-mediated delivery markedly enhanced the uniformity and persistence of intradermal drug distribution. In the rabbit ear HS model, treatment with sEVs^ErF^-loaded DMNPs subsequently reduced the SEI and promoted the restoration of a more physiological collagen architecture, accompanied by biochemical markers of ferroptosis activation and transcriptomic signatures indicative of constructive tissue remodeling. The biocompatibility of the microneedle materials is well established: GelMA is derived from natural gelatin and has been extensively utilized in tissue engineering and drug delivery [52], while PVA is an FDA-approved polymer with a well-documented safety profile [53]. In our study, the absence of local inflammation and systemic abnormalities further confirm the safety of this platform. Collectively, these data indicate that our sEVs^ErF^-DMNP platform is well-tolerated and suitable for further translational development.

This study has several limitations. First, while our competitive blocking and FAP knockdown experiments confirmed FAP-specific targeting, additional validation using FAP knockout models and testing across multiple donor-derived primary cells would further strengthen generalizability. Second, the pharmacokinetic analysis relied on fluorescence-based tracking, which does not provide absolute quantification of sEV concentration in tissue. Future work should incorporate time-resolved uptake, broader off-target evaluations across additional scar-resident cell types, and larger cohorts with longer-term safety assessments. These aspects represent important directions for future investigation.

## 5 Conclusion

In conclusion, this study introduces a robust and versatile platform for precision scar therapy through the integration of FAP-targeted and erastin-loaded sEVs with a dissolvable microneedle patch. This system harmonizes three synergistic therapeutic attributes: molecular specificity via FAP-directed targeting, ferroptosis-based inactivation of HSFs, and spatiotemporal control via microneedle-enabled localized delivery. The modular nature of this platform enables future adaptation through the exchange of therapeutic cargoes or biomaterial elements, thereby markedly expanding its potential utility. Moreover, this strategy holds promise not only for fibroblast-driven conditions such as keloids and organ fibrosis, but also for certain FAP-rich solid tumors, this remains to be established in dedicated tumor models [54]. While additional validation using human-derived models and comprehensive safety profiling will be crucial for clinical progression, the present work establishes a compelling and adaptable framework for achieving cell-specific and sustained intervention across a wide range of FAP-associated diseases.

## Funding

This work was supported by the 10.13039/501100012166National Key Research and Development Program of China (2022YFA1104300); the 10.13039/501100001809National Natural Science Foundation of China (82172211, 92268206, 92468303); and the 10.13039/501100005089Natural Science Foundation of Beijing (7242129).

## CRediT authorship contribution statement

**Yangmengyuan Xu:** Conceptualization, Data curation, Investigation, Methodology, Validation, Visualization, Writing – original draft. **Yiqing Zhang:** Data curation, Investigation, Methodology, Visualization, Writing – original draft. **Zijie Sun:** Data curation, Investigation, Methodology, Validation. **Junfeng Gong:** Data curation, Methodology, Validation. **Qi Shen:** Data curation, Validation. **Hao Meng:** Investigation, Resources. **Xi Liu:** Investigation, Resources. **Junli Chen:** Investigation, Resources. **Yaying Hao:** Investigation, Resources. **Zhan Xu:** Project administration, Resources. **Kui Ma:** Resources, Supervision. **Liqian Ma:** Project administration, Resources. **Kailu Guo:** Visualization. **Xiaohua Pan:** Conceptualization, Resources, Supervision, Writing – review & editing. **Xiaobing Fu:** Conceptualization, Funding acquisition, Project administration, Resources, Supervision, Writing – review & editing. **Cuiping Zhang:** Conceptualization, Funding acquisition, Project administration, Resources, Supervision, Writing – review & editing.

## Declaration of competing interest

The authors declare that they have no known competing financial interests or personal relationships that could have appeared to influence the work reported in this paper.

## Data Availability

Data will be made available on request.

## References

[1] Jeschke M.G., Wood F.M., Middelkoop E., Bayat A., Teot L., Ogawa R., Gauglitz G.G. (2023). Scars. Nat. Rev. Dis. Primers.

[2] Frech F.S., Hernandez L., Urbonas R., Zaken G.A., Dreyfuss I., Nouri K. (2023). Hypertrophic scars and keloids: advances in treatment and review of established therapies. Am. J. Clin. Dermatol..

[3] Fang X., Wang Y., Chen H., Yan Z., Jin S., Wu Y., Shu F., Xiao S. (2025). Hypertrophic scarring and keloids: epidemiology, molecular pathogenesis, and therapeutic interventions. MedComm.

[4] Shook B.A., Wasko R.R., Rivera-Gonzalez G.C., Salazar-Gatzimas E., López-Giráldez F., Dash B.C., Muñoz-Rojas A.R., Aultman K.D., Zwick R.K., Lei V., Arbiser J.L., Miller-Jensen K., Clark D.A., Hsia H.C., Horsley V. (2018). Myofibroblast proliferation and heterogeneity are supported by macrophages during skin repair. Science.

[5] He J., Fang B., Shan S., Xie Y., Wang C., Zhang Y., Zhang X., Li Q. (2021). Mechanical stretch promotes hypertrophic scar formation through mechanically activated cation channel Piezo1. Cell Death Dis..

[6] Talbott H.E., Mascharak S., Griffin M., Wan D.C., Longaker M.T. (2022). Wound healing, fibroblast heterogeneity, and fibrosis. Cell Stem Cell.

[7] Dixon S.J., Lemberg K.M., Lamprecht M.R., Skouta R., Zaitsev E.M., Gleason C.E., Patel D.N., Bauer A.J., Cantley A.M., Yang W.S., Morrison B., Stockwell B.R. (2012). Ferroptosis: an iron-dependent form of nonapoptotic cell death. Cell.

[8] Zhao W.G., Zhou X., Xue Y., Wang T., Li Q., Tan L.-L., Shang L. (2023). Ferroptosis-mediated synergistic therapy of hypertrophic scarring based on metal-organic framework microneedle patch. Adv. Funct. Mater..

[9] Chen Y., Wang S., Mao C., Lu Q., Zhu X., Fan D., Liu Y., Chen X., Zhan J., Yang Z., Ji P., He Q., Chen T. (2025). 5-ALA photodynamic metabolite-powered zero-waste ferroptosis amplifier for enhanced hypertrophic scar therapy. Nat. Commun..

[10] Xie Y., Hou W., Song X., Yu Y., Huang J., Sun X., Kang R., Tang D. (2016). Ferroptosis: process and function. Cell Death Differ..

[11] Sun S., Shen J., Jiang J., Wang F., Min J. (2023). Targeting ferroptosis opens new avenues for the development of novel therapeutics. Signal Transduct. Targeted Ther..

[12] Sun Z., Zheng Q., Zhang Y. (2025). Hydrogel loaded with aminoethyl anisamide-modified exosomes attenuates hepatic fibrosis by targeting activated hepatic stellate cells. ACS Nano.

[13] Jang H., Park D., Park B. (2025). Oral PTP1B siRNA delivery using milk-derived extracellular vesicles for alleviation of acute kidney injury. ACS Nano.

[14] Théry C., Witwer K.W., Aikawa E., Alcaraz M.J., Anderson J.D., Andriantsitohaina R., Antoniou A., Arab T., Archer F., Atkin-Smith G.K. (2018). Minimal information for studies of extracellular vesicles 2018 (MISEV2018): a position statement of the international society for extracellular vesicles and update of the MISEV2014 guidelines. J. Extracell. Vesicles.

[15] Zhang M., Hu S., Liu L., Dang P., Liu Y., Sun Z., Qiao B., Wang C. (2023). Engineered exosomes from different sources for cancer-targeted therapy. Signal Transduct. Targeted Ther..

[16] Fitzgerald A.A., Weiner L.M. (2020). The role of fibroblast activation protein in health and malignancy. Cancer Metastasis Rev..

[17] Jansen K., Heirbaut L., Verkerk R., Cheng J.D., Joossens J., Cos P., Maes L., Lambeir A.M., De Meester I., Augustyns K., Van der Veken P. (2014). Extended structure-activity relationship and pharmacokinetic investigation of (4-quinolinoyl)glycyl-2-cyanopyrrolidine inhibitors of fibroblast activation protein (FAP). J. Med. Chem..

[18] Neri-Cruz C.E., Teixeira F.M.E., Gautrot J.E. (2023). A guide to functionalisation and bioconjugation strategies to surface-initiated polymer brushes. Chem. Commun..

[19] Liu Q., Li D., Pan X., Liang Y. (2023). Targeted therapy using engineered extracellular vesicles: principles and strategies for membrane modification. J. Nanobiotechnol..

[20] Han X., Saengow C., Ju L., Ren W., Ewoldt R.H., Irudayaraj J. (2024). Exosome-coated oxygen nanobubble-laden hydrogel augments intracellular delivery of exosomes for enhanced wound healing. Nat. Commun..

[21] Prausnitz M.R., Langer R. (2008). Transdermal drug delivery. Nat. Biotechnol..

[22] Zhao E., Xiao T., Tan Y., Zhou X., Li Y., Wang X., Zhang K., Ou C., Zhang J., Li Z., Liu H. (2023). Separable microneedles with photosynthesis-driven oxygen manufactory for diabetic wound healing. ACS Appl. Mater. Interfaces.

[23] Meng S., Wei Q., Chen S., Liu X., Cui S., Huang Q., Chu Z., Ma K., Zhang W., Hu W., Li S., Wang Z., Tian L., Zhao Z., Li H., Fu X., Zhang C. (2024). MiR-141-3p-functionalized exosomes loaded in dissolvable microneedle arrays for hypertrophic scar treatment. Small.

[24] Vorstandlechner V., Laggner M., Copic D., Klas K., Direder M., Chen Y., Golabi B., Haslik W., Radtke C., Tschachler E., Hötzenecker K., Ankersmit H.J., Mildner M. (2021). The serine proteases dipeptidyl-peptidase 4 and urokinase are key molecules in human and mouse scar formation. Nat. Commun..

[25] Tedeschini T., Campara B., Grigoletto A., Bellini M., Salvalaio M., Matsuno Y., Suzuki A., Yoshioka H., Pasut G. (2021). Polyethylene glycol-based linkers as hydrophilicity reservoir for antibody-drug conjugates. J. Contr. Release.

[26] Li M., Younis M.H., Zhang Y., Cai W., Lan X. (2022). Clinical summary of fibroblast activation protein inhibitor-based radiopharmaceuticals: cancer and beyond. Eur. J. Nucl. Med. Mol. Imag..

[27] Hlavackova A., Vydra J., Chrastinova L., Kotlin R., Stikarova J., Suttnar J., Dyr J. (2019). Alteration of serum malondialdehyde level as biomarker of oxidative stress during acute myeloid leukemia treatment. Blood.

[28] Cao J.Y., Dixon S.J. (2016). Mechanisms of ferroptosis. Cell. Mol. Life Sci..

[29] Yan H.F., Zou T., Tuo Q.Z., Xu S., Li H., Belaidi A.A., Lei P. (2021). Ferroptosis: mechanisms and links with diseases. Signal Transduct. Targeted Ther..

[30] Huang W., Zhang Y., Das N.K., Solanki S., Jain C., El-Derany M.O., Koo I., Bell H.N., Aabed N., Singhal R., Castillo C., Buscher K., Ying Y., Dimitroff J., Sharma A., Shi J., Hogan S.P., Dame M.K., Higgins P.D.R., Colacino J.A., Oh T.G., Spence J.R., Patterson A.D., Greenberg A.S., Greenson J.K., Nusrat A., Shah Y.M. (2025). Fibroblast lipid metabolism through ACSL4 regulates epithelial sensitivity to ferroptosis in IBD. Nat. Metab..

[31] Ghosh S., Ghosh S., Yeung K.W.K., Xu C., Wu J., Mao C., Ling Z., Okuro K., Qiao W. (2025). Dual-responsive copper complex delivered by dissolvable microneedle promotes polymicrobial infection eradication and cutaneous wound healing. Mater. Today Bio.

[32] Saifullah K.M., Mushtaq A., Azarikhah P., Prewett P.D., Davies G.J., Faraji Rad Z. (2025). Micro-vibration assisted dual-layer spiral microneedles to rapidly extract dermal interstitial fluid for minimally invasive detection of glucose. Microsyst. Nanoeng..

[33] Kloeters O., Tandara A., Mustoe T.A. (2007). Hypertrophic scar model in the rabbit ear: a reproducible model for studying scar tissue behavior with new observations on silicone gel sheeting for scar reduction. Wound Repair Regen..

[34] Branyiczky M.K., Metko D., Hsu J.T.S., Li M.K. (2025). Combination of carbon dioxide laser and triamcinolone acetonide for keloid and hypertrophic scar treatment: a systematic review. J. Am. Acad. Dermatol..

[35] Younas A., Asad M., Wan X., Zhang Y., Ma X., Wang L., Gu H., Shang H., Zhang N. (2024). Oregano essential oil-infused mucin microneedle patch for the treatment of hypertrophic scar. Int. J. Pharm..

[36] Wynn T.A., Ramalingam T.R. (2012). Mechanisms of fibrosis: therapeutic translation for fibrotic disease. Nat. Med..

[37] Yang W.S., SriRamaratnam R., Welsch M.E., Shimada K., Skouta R., Viswanathan V.S., Cheah J.H., Clemons P.A., Shamji A.F., Clish C.B., Brown L.M., Girotti A.W., Cornish V.W., Schreiber S.L., Stockwell B.R. (2014). Regulation of ferroptotic cancer cell death by GPX4. Cell.

[38] Doll S., Proneth B., Tyurina Y.Y., Panzilius E., Kobayashi S., Ingold I., Irmler M., Beckers J., Aichler M., Walch A., Prokisch H., Trümbach D., Mao G., Qu F., Bayir H., Füllekrug J., Scheel C.H., Wurst W., Schick J.A., Kagan V.E., Angeli J.P.F., Conrad M. (2017). ACSL4 dictates ferroptosis sensitivity by shaping cellular lipid composition. Nat. Chem. Biol..

[39] Henderson N.C., Rieder F., Wynn T.A. (2020). Fibrosis: from mechanisms to medicines. Nature.

[40] Anandhan A., Dodson M., Shakya A., Chen J., Liu P., Wei Y., Tan H., Wang Q., Jiang Z., Yang K., Garcia J.G.N., Chambers S.K., Chapman E., Ooi A., Yang-Hartwich Y., Stockwell B.R., Zhang D.D. (2023). NRF2 controls iron homeostasis and ferroptosis through HERC2 and VAMP8. Sci. Adv..

[41] Chao H., Zheng L., Hsu P., He J., Wu R., Xu S., Zeng R., Zhou Y., Ma H., Liu H., Tang Q. (2023). IL-13RA2 downregulation in fibroblasts promotes keloid fibrosis via JAK/STAT6 activation. JCI Insight.

[42] Gandham S., Su X., Wood J., Nocera A.L., Alli S.C., Milane L., Zimmerman A., Amiji M., Ivanov A.R. (2020). Technologies and standardization in research on extracellular vesicles. Trends Biotechnol..

[43] Wang J., Yu N., Wang G. (2023). 68Ga-FAPI-04 PET/CT in assessment of fibroblast activation in keloids: a prospective pilot study. Clin. Nucl. Med..

[44] Zhou P., Ding X., Du X., Wang L., Zhang Y. (2024). Targeting reprogrammed cancer-associated fibroblasts with engineered mesenchymal stem cell extracellular vesicles for pancreatic cancer treatment. Biomater. Res..

[45] Wang Y., Jiang H., Chen Q., Guo F., Zhang B., Hu L., Huang X., Shen W., Gao J., Chen W., Xu W., Cai Z., Wei L., Li M. (2025). Myofibroblast-targeting extracellular vesicles: a promising platform for cardiac fibrosis drug delivery. Biomater. Res..

[46] Chen X., Kang R., Kroemer G., Tang D. (2021). Broadening horizons: the role of ferroptosis in cancer. Nat. Rev. Clin. Oncol..

[47] Weiland A., Wang Y., Wu W., Lan X., Han X., Li Q., Wang J. (2019). Ferroptosis and its role in diverse brain diseases. Mol. Neurobiol..

[48] Lai W., Wang B., Huang R., Zhang C., Fu P., Ma L. (2024). Ferroptosis in organ fibrosis: from mechanisms to therapeutic medicines. J. Transl. Intern. Med..

[49] Xue H., Zhang C., Lin D., Gu Q., Sun C., Lin X., Zhang C., Lei L., Liu L. (2025). Isoliquiritigenin micellar microneedle for pH monitoring and diabetic wound healing. Mater. Today Bio.

[50] Li Y., Chen K., Pang Y. (2023). Multifunctional microneedle patches via direct ink drawing of nanocomposite inks for personalized transdermal drug delivery. ACS Nano.

[51] Yan D., Cao G., Gao Y., Wang Y., Zhang W., Wang K., Mao S., Li C., Zhou G., Xia H., Dai W., Yan X., Wang Y. (2025). Photothermal-controlled microneedle for transdermal delivery of metal-phenolic nanozyme with staged multifunctions to accelerate healing of infected diabetic wounds. Mater. Today Bio.

[52] Zhang Y., Li H., Li G., Chen Y., Zeng Y. (2025). Hydrogel-forming microneedles for the treatment of skin diseases. Mater. Today Bio.

[53] Shen S., Shen W., Wang L., Sun B., Zhang Y., Zhang Y., Jia R., Wu Y., Chen X., Cao K., Fang Y., Xia H. (2025). Berberine hydrochloride-loaded liposomes-in-hydrogel microneedles achieve the efficient treatment for psoriasis. Mater. Today Bio.

[54] Hagens M.J., van Leeuwen P.J., Wondergem M., Boellaard T.N., Sanguedolce F., Oprea-Lager D.E., Bex A., Vis A.N., van der Poel H.G., Mertens L.S. (2024). A systematic review on the diagnostic value of fibroblast activation protein inhibitor PET/CT in genitourinary cancers. J. Nucl. Med..

