# Supplementary material for: Rational design of FAP-targeted sEVs delivered by microneedles for precision treatment of hypertrophic scars via ferroptosis in hypertrophic scar fibroblasts
Source: Mater Today Bio. 2026 Apr 13;38:103117. doi: 10.1016/j.mtbio.2026.103117 (PMC13098611; doi:10.1016/j.mtbio.2026.103117)
Supplement: Multimedia component 1 [file mmc1.docx]

Supplementary file

**
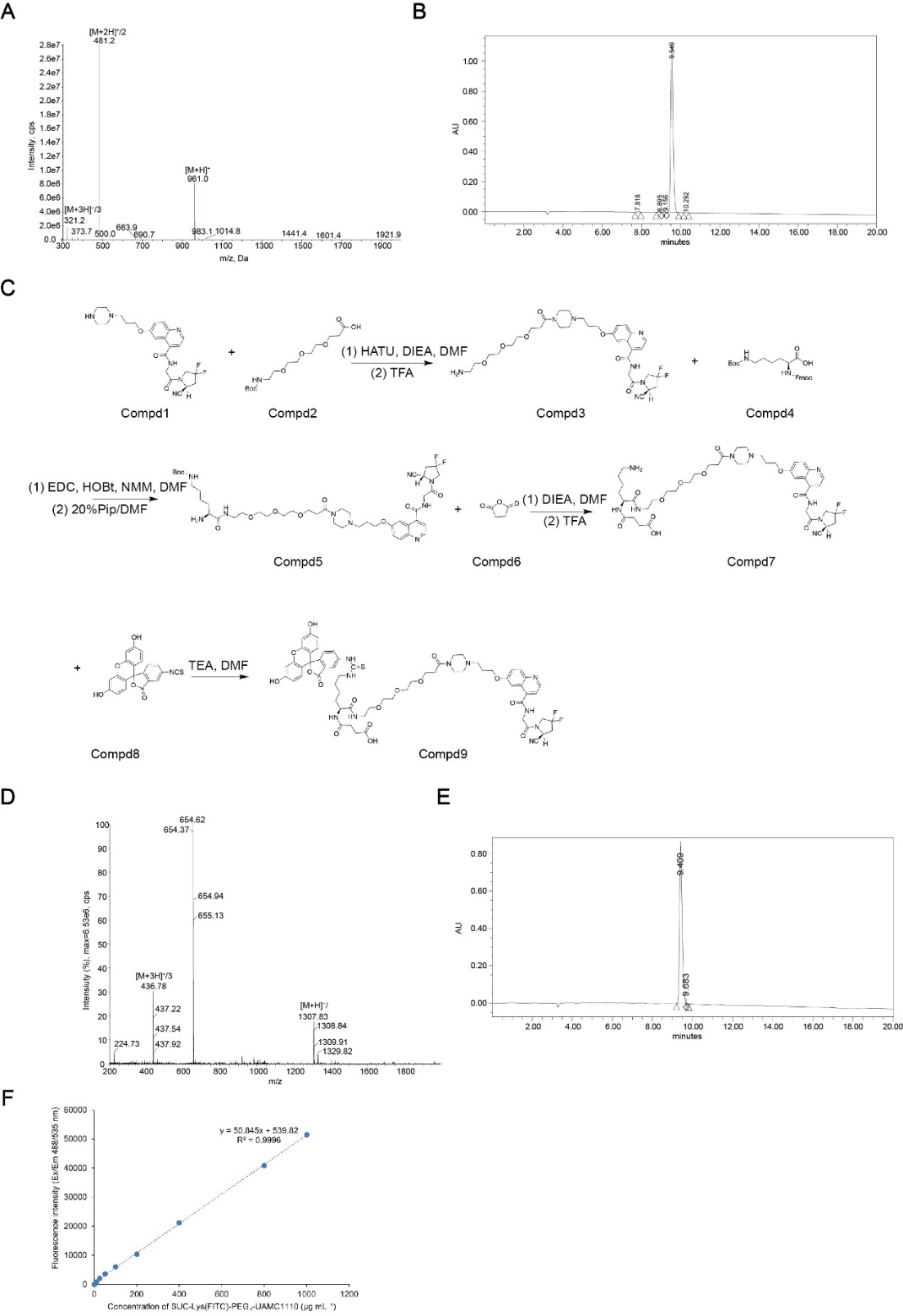
**

**Fig. S1.** Chemical identity and quantitative labeling of FAP-targeting ligands. (A) HPLC chromatogram of SUC-Lys(Ac)-PEG₃-UAMC1110 (purity ~99.78%). (B) Mass spectrum of SUC-Lys(Ac)-PEG₃-UAMC1110. (C) Synthetic scheme of SUC-Lys(FITC)-PEG₃-UAMC1110. (D) HPLC chromatogram of SUC-Lys(FITC)-PEG₃-UAMC1110 (purity ~98.52%). (E) Mass spectrum corresponding to panel D. (F) Standard fluorescence calibration curve for SUC-Lys(FITC)-PEG₃-UAMC1110 (R² = 0.9996); estimated ligand density ~2.25 × 10⁴ small molecules per sEV.


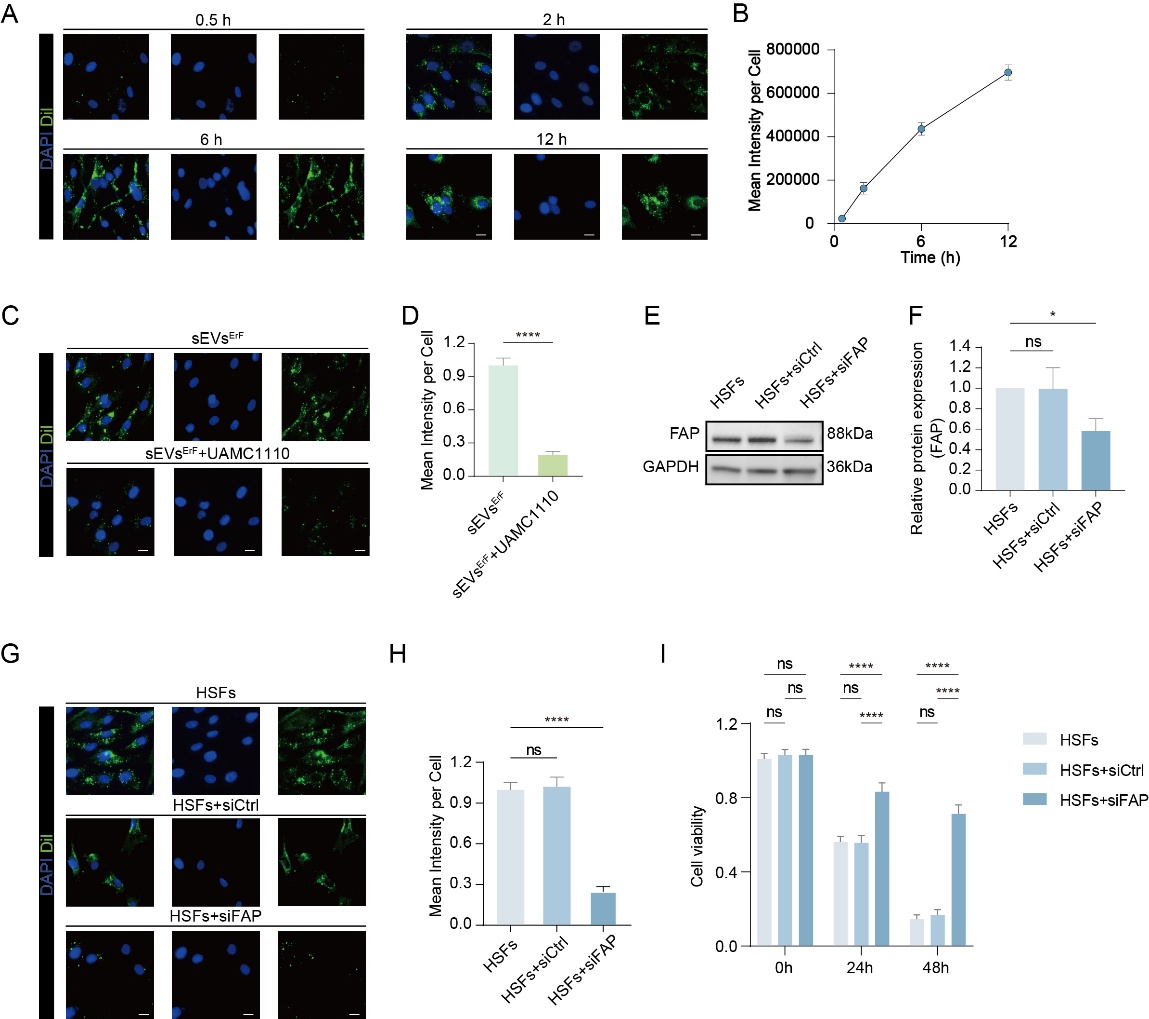


**Fig. S2. Uptake kinetics and FAP-specificity of sEVs^ErF^ in HSFs.** (A) Representative fluorescence images of HSFs incubated with FITC-labeled sEVs^ErF^ for 0.5, 2, 6, and 12 h. Green, sEVs^ErF^-FITC; blue, DAPI. Scale bar, 10 μm. (B) Quantification of mean fluorescence intensity per cell at each time point (n = 5), showing progressive increase in uptake over time. (C) Representative images of HSFs incubated with FITC-labeled sEVs^ErF^ for 2 h with or without pretreatment with free UAMC1110 as a competitor. Scale bar, 10 μm. (D) Quantification of cellular fluorescence intensity relative to the non-blocked group (n = 5). Data are presented as mean ± SD. ****p < 0.0001. (E) Western blot analysis of FAP expression in untransfected HSFs, HSFs transfected with control siRNA (siCtrl), and HSFs transfected with FAP-specific siRNA (siFAP). GAPDH served as loading control. (F) Quantification of FAP protein expression relative to GAPDH (n = 3). (G) Representative fluorescence images of untransfected HSFs, siCtrl-transfected HSFs, and siFAP-transfected HSFs incubated with FITC-labeled sEVs^ErF^ for 2 h. Scale bar, 10 μm. (H) Quantification of cellular fluorescence intensity relative to untransfected group (n = 5). (I) CCK-8 assay of HSF viability after 24 h or 48 h treatment with sEVs^ErF^ in untransfected HSFs, siCtrl-transfected HSFs, and siFAP-transfected HSFs. Data are mean ± SD (n = 6). *p < 0.05, ****p < 0.0001; ns, not significant.


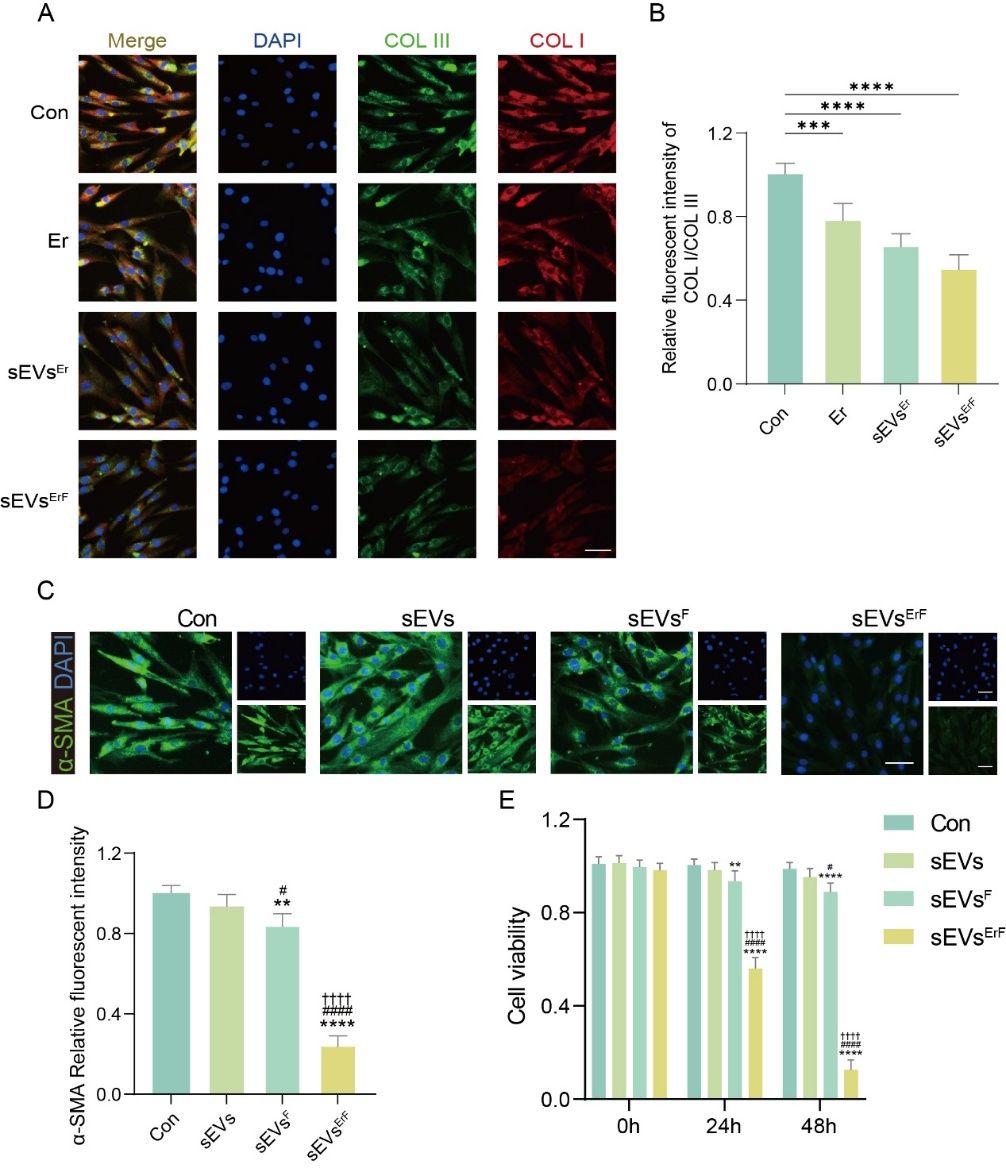


**Fig. S3**. (A) Representative immunofluorescence images of HSFs after treatment with Control (Con), Er, sEVs^Er^, or sEVs^ErF^. Type I collagen (COL I, red), type III collagen (COL III, green), and nuclei (DAPI, blue). Scale bar, 50 μm. (B) Quantification of the COL I/COL III fluorescence-intensity ratio (n = 5). ***p < 0.001; ****p < 0.0001. (C) Representative immunofluorescence images of α-SMA (green) in HSFs after treatment with PBS, unmodified sEVs, sEVs^F^ (UAMC1110-modified sEVs without erastin), or sEVs^ErF^. Nuclei were stained with DAPI (blue). Scale bar, 50 μm. (D) Quantification of α-SMA fluorescence intensity relative to PBS control (n = 5). (E) CCK-8 assay of HSF viability at 24 h and 48 h after indicated treatments. Data are presented as mean ± SD (n = 6). **p < 0.01, ****p < 0.0001 vs Con; #p < 0.05, ####p < 0.0001 vs sEVs; ††††p < 0.0001 vs sEVs^F^.


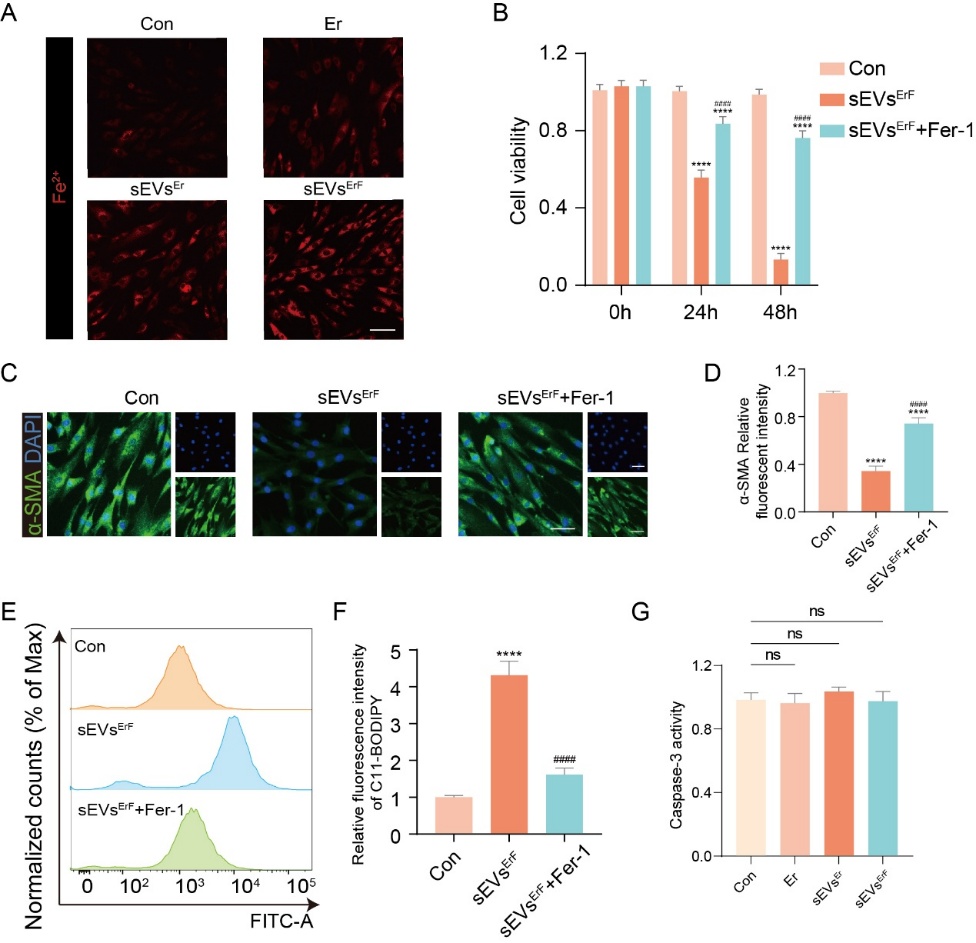


**Fig. S4.** (A) Representative fluorescence micrographs of HSFs after the indicated treatments (Con, Er, sEVs^Er^, sEVs^ErF^) stained with FerroOrange to visualize labile Fe²⁺. Scale bar, 50 μm. Quantification of relative Fe²⁺ fluorescence intensity is shown in Fig. 4F (n = 5). (B) CCK-8 assay of HSF viability at 24 h and 48 h after treatment with Control, sEVs^ErF^, or sEVs^ErF^ + Fer-1. (n = 6) (C) Representative immunofluorescence images of α-SMA (green) in HSFs after 24 h treatment. Nuclei were stained with DAPI (blue). Scale bar, 50 μm. (D) Quantification of α-SMA fluorescence intensity relative to Control. (n = 5) (E) Representative flow cytometry plots of C11-BODIPY 581/591 staining for lipid ROS. (F) Quantification of lipid ROS fluorescence intensity relative to Control. Data are presented as mean ± SD (n = 3). ****p < 0.0001 vs Control; ####p < 0.0001 vs sEVs^ErF^.  (G) Caspase-3 activity in HSFs treated with Control, Er, sEVs^Er^, or sEVs^ErF^ for 24 h. Data are presented as mean ± SD (n = 3). ns, not significant.


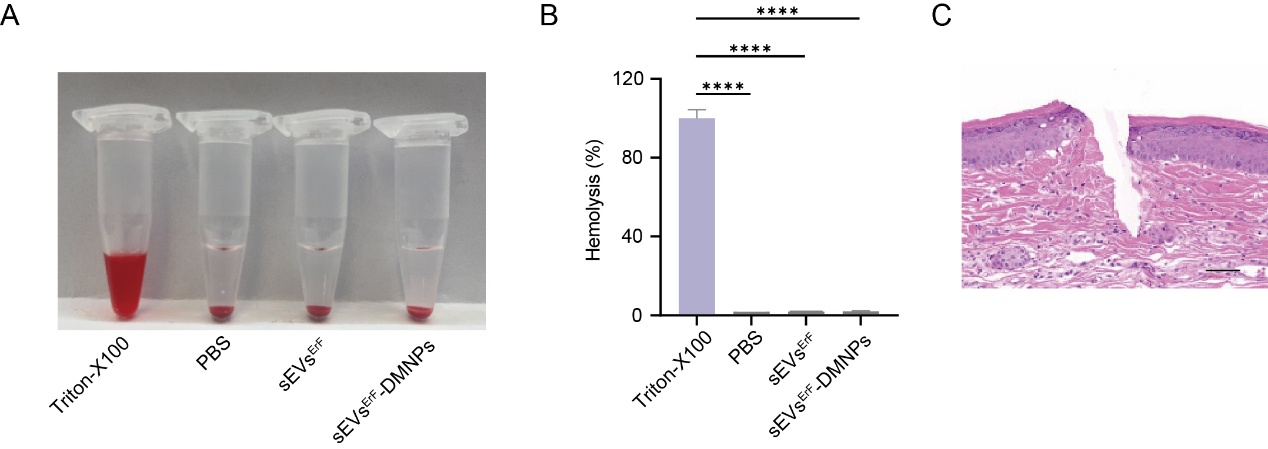


**Fig. S5.** Hemolysis evaluation of sEVs^ErF^ and sEVs^ErF^-DMNP. (A) Representative images of red blood cell (RBC) suspensions after incubation with Triton X‑100 (positive control), PBS (negative control), sEVs^ErF^, or sEVs^ErF^-DMNP. (B) Quantitative analysis of hemolysis percentage, calculated based on absorbance at 540 nm. Hemolysis (%) was determined using the following formula:

  Hemolysis (%) = (OD_sample_ − OD_PBS_) / (OD_Triton_ − OD_PBS_) × 100%.

The hemolysis rates were ~0.6% for sEVs^ErF^ and ~2.04% for sEVs^ErF^-DMNP (n = 3 per group). (C) Representative H&E staining of rabbit ear skin after DMNPs application, showing microneedle penetration depth (arrows indicate insertion tracks). Scale bar, 100 μm.


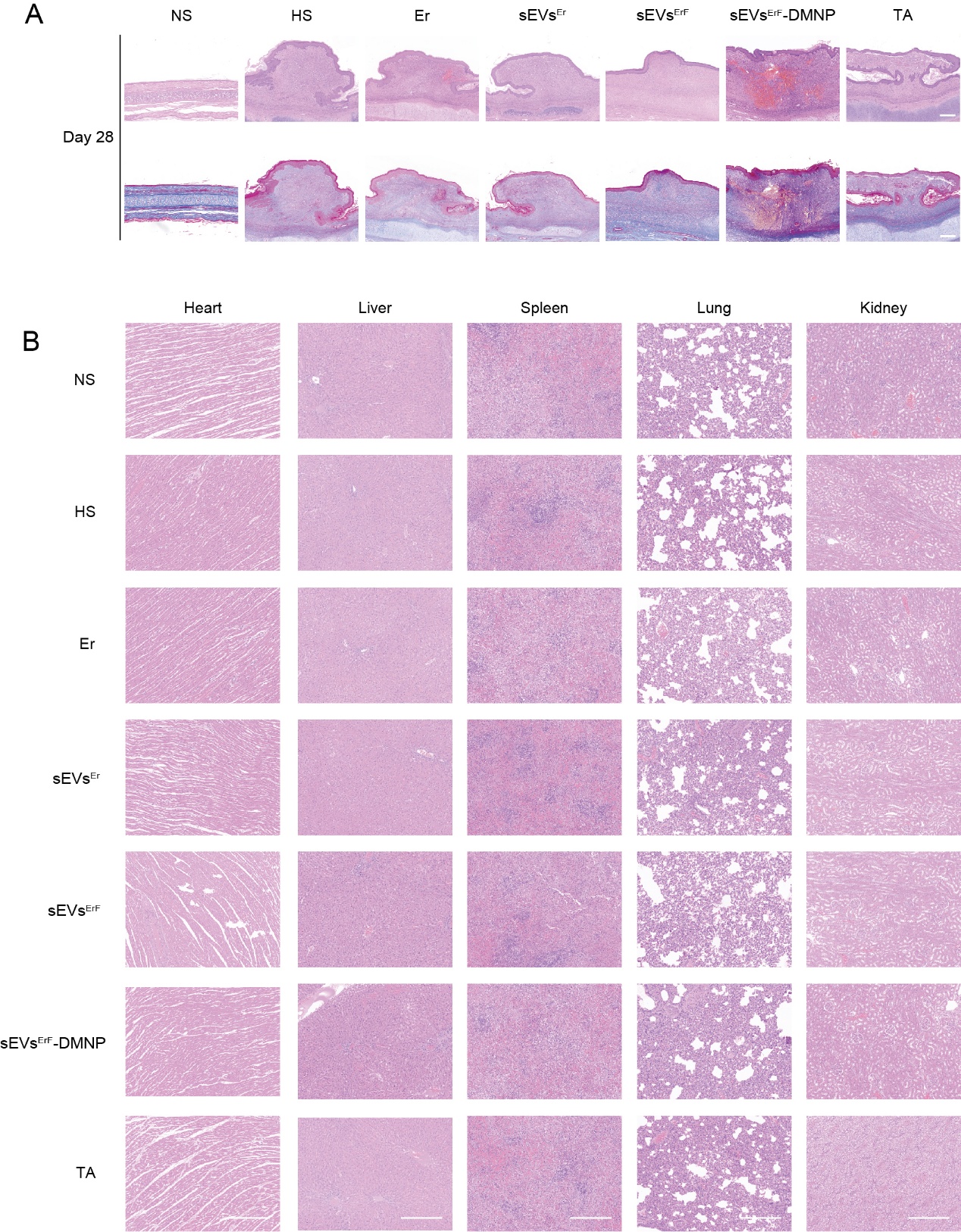


**Fig. S6.** Baseline histology and systemic safety. (A) Baseline (day 28, pretreatment) histology of hypertrophic scars showing thickened dermis and densely packed, disorganized collagen on H&E and Masson’s trichrome, confirming the HS phenotype prior to intervention. Scale bar, 400 μm. (B) Representative H&E of major organs (heart, liver, spleen, lung, kidney) at day 49 across groups, showing no overt pathological changes. Scale bar, 400 μm.


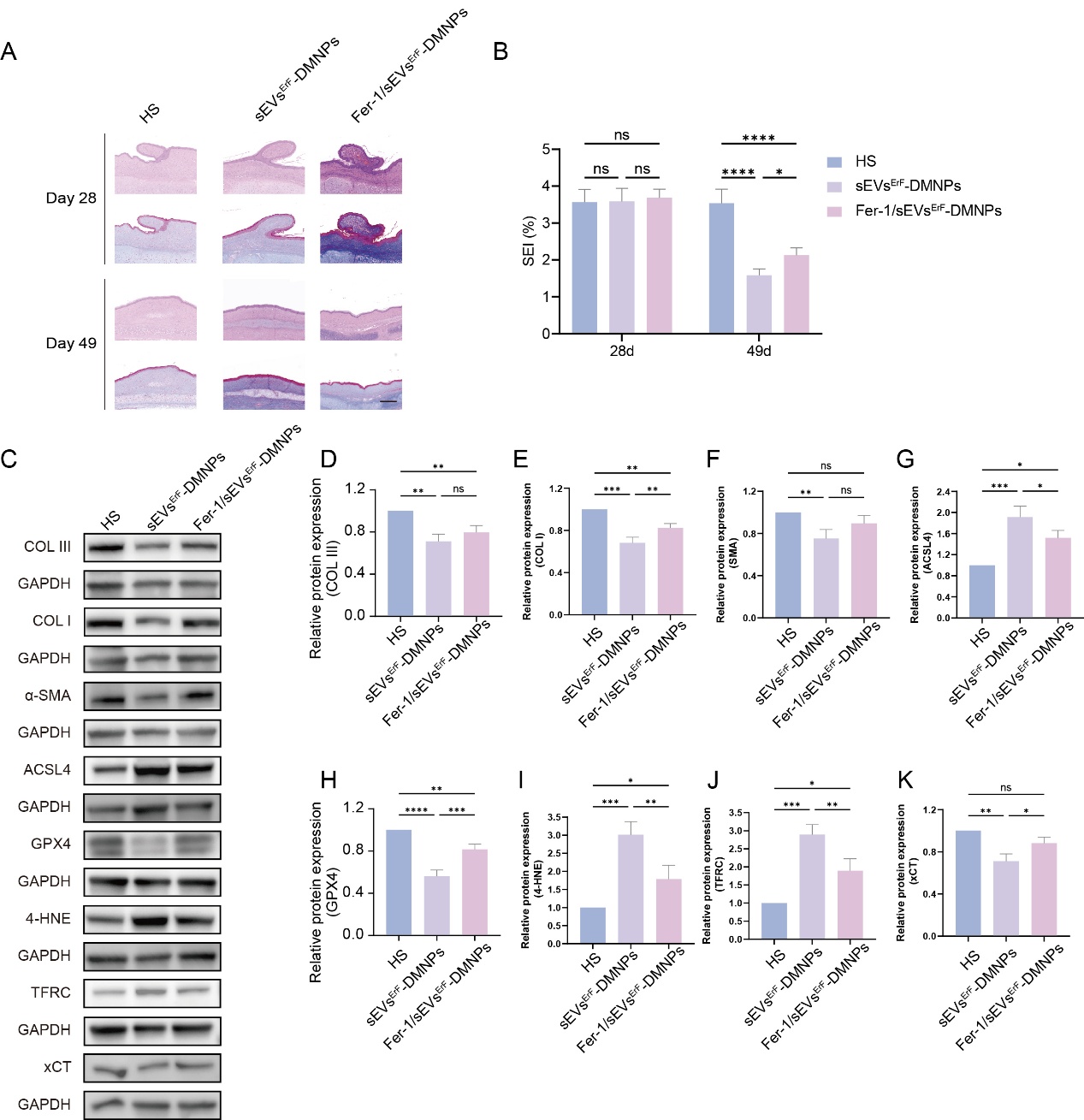


**Fig. S7.** (A) Representative H&E staining of scar tissues from HS, sEVs^ErF^-DMNPs, and Fer-1/sEVs^ErF^-DMNPs groups at day 28 and 49. Scale bar, 500 μm. (B) Quantification of SEI at day 28 and 49. (C) Western blot analysis of COL I, COL III, α-SMA, GPX4, ACSL4, xCT, TFRC, and 4-HNE in scar tissues from the three groups. GAPDH served as loading control. (D-K) Densitometric quantification of protein expression relative to GAPDH. Data are presented as mean ± SD (n = 3 per group). *p < 0.05, **p < 0.01, ***p < 0.001, ****p < 0.0001.

**
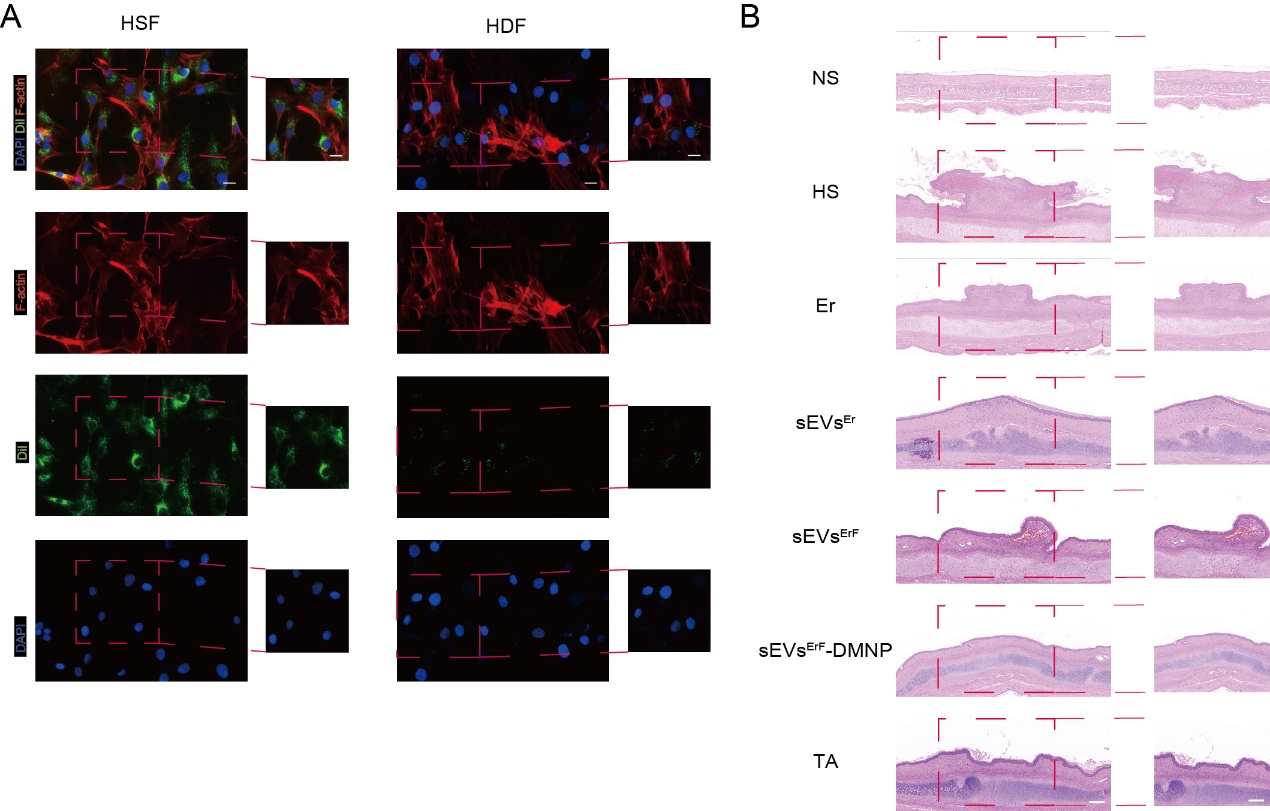
**

**Fig. S8. Representative raw images and ROI selection for image quantification.**
(A) Representative raw fluorescence image of cellular uptake (FITC-labeled sEVs^ErF^ in HSFs) with the region of interest (ROI) outlined, showing the entire field of view used for quantification. Scale bar, 10 μm. (B) Representative raw H&E-stained section of scar tissue with the ROI outlined, indicating the dermal scar region selected for histological quantification. Scale bar, 400 μm.

**Table S1. Hematological parameters at day 49.**

| Groups | Value | | | | | |
| --- | --- | --- | --- | --- | --- | --- |
|  | WBC (*10^9^/L) | RBC (*10^12^/L) | HGB (g/L) | HCT (%) | MCV (%) | PLT (*10^9^/L) |
| NS | 5.58 ± 0.46 | 4.77 ± 0.38 | 111.01 ± 5.65 | 41.13 ± 1.52 | 65.22 ± 3.20 | 131.84 ± 16.28 |
| HS | 5.45 ± 0.33 | 5.79 ± 0.59 | 120.37 ± 6.20 | 40.33 ± 3.52 | 66.18 ± 0.85 | 130.93 ± 18.88 |
| Er | 4.82 ± 0.96 | 5.28 ± 0.53 | 121.90 ± 9.40 | 37.01 ± 1.63 | 64.09 ± 2.78 | 149.78 ± 10.27 |
| sEVs^Er^ | 5.51 ± 0.20 | 4.65 ± 0.19 | 125.62 ± 12.64 | 35.95 ± 3.54 | 66.81 ± 3.37 | 123.04 ± 33.00 |
| sEVs^ErF^ | 4.91 ± 0.67 | 4.64 ± 0.27 | 117.83 ± 12.61 | 39.07 ± 3.12 | 63.49 ± 1.87 | 106.78 ± 19.75 |
| sEVs^ErF^-DMNPs | 5.01 ± 0.76 | 5.19 ± 0.61 | 122.46 ± 4.15 | 40.43 ± 2.51 | 63.49 ± 1.72 | 113.85 ± 23.92 |
| TA | 5.24 ± 0.45 | 5.44 ± 0.56 | 127.72 ± 10.97 | 33.78 ± 2.90 | 66.61 ± 3.32 | 145.46 ± 22.31 |

**Table S2. Serum biochemical parameters at day 49.**

| Groups | Value | | | | |
| --- | --- | --- | --- | --- | --- |
|  | ALT (U/L) | AST (U/L) | ALP (U/L) | UREA (mmol/L) | CREA (umol/L) |
| NS | 17.86 ± 6.97 | 36.83 ± 7.82 | 38.90 ± 8.33 | 4.97 ± 0.28 | 31.88 ± 3.20 |
| HS | 25.59 ± 0.91 | 35.37 ± 3.87 | 36.29 ± 6.14 | 5.09 ± 0.27 | 37.31 ± 4.26 |
| Er | 20.27 ± 6.69 | 41.97 ± 4.07 | 44.49 ± 3.72 | 4.72 ± 0.32 | 41.23 ± 6.50 |
| sEVs^Er^ | 24.62 ± 1.50 | 44.85 ± 2.31 | 41.33 ± 0.97 | 5.18 ± 0.28 | 37.13 ± 2.75 |
| sEVs^ErF^ | 23.25 ± 3.20 | 34.82 ± 4.04 | 38.67 ± 4.16 | 4.80 ± 0.19 | 36.74 ± 5.78 |
| sEVs^ErF^-DMNPs | 19.94 ± 2.84 | 37.18 ± 6.82 | 36.89 ± 6.83 | 5.11 ± 0.14 | 38.58 ± 5.94 |
| TA | 16.02 ± 3.21 | 34.72 ± 2.45 | 38.04 ± 4.86 | 5.08 ± 0.34 | 42.19 ± 4.37 |

**Table S3. Pharmacokinetic comparison of sEVs^ErF^ delivered by injection versus DMNPs.**

| **Parameter** | **Intradermal injection** | **DMNPs** |
| --- | --- | --- |
| Cmax (normalized) | 100% | 100% |
| Residence half-life (t1/2, days) | 1.0 | 1.9 |
| AUC0–7d (normalized, %·day) | 129 | 259 |
